# Supplementary material for: ALS-associated RNA-binding proteins promote UNC13A transcription through REST downregulation
Source: EMBO J. 2025 Jul 24;44(17):4745–71. doi: 10.1038/s44318-025-00506-0 (PMC12402202; doi:10.1038/s44318-025-00506-0)
Supplement: Supplementary file 3 — Appendix [file 44318_2025_506_MOESM3_ESM.pdf]

Appendix for

ALS-associated RNA-binding proteins promote *UNC13A* transcription through REST downregulation

Watanabe *et al*.

Appendix Figure S1…. Page 2

Appendix Figure S2…. Page 3

Appendix Figure S3…. Page 4,5

Appendix Figure S4…. Page 6,7

Appendix Figure S5…. Page 8

Appendix Figure S6…. Page 9

Appendix Figure S7…. Page 10,11

Appendix Table S1…. Page 12

Appendix Table S2…. Page 13

Appendix Table S3…. Page 14

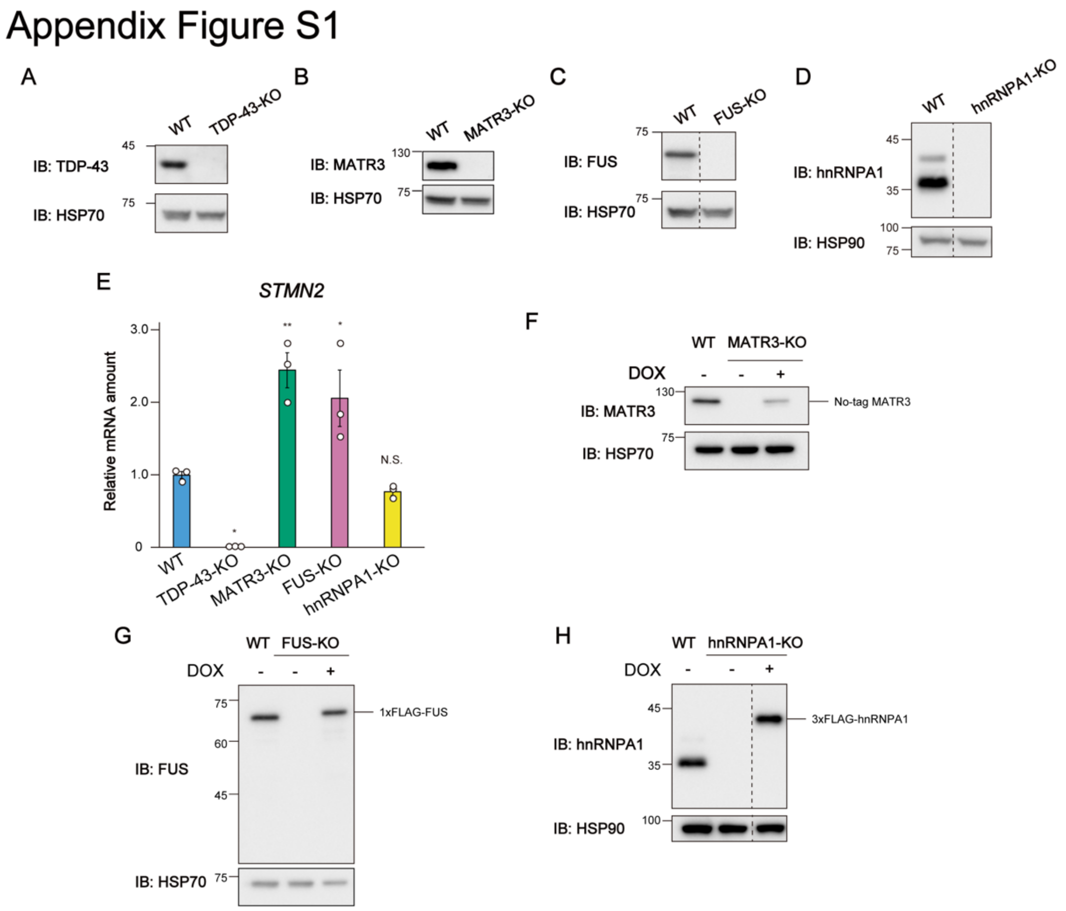

Appendix Figure S1. Generation of RBP-KO cell lines and rescue of corresponding RBP expression, related to Figure 1

(A–D) Immunoblot (IB) analysis of RBPs in WT and TDP-43-KO (A), MATR3-KO (B), FUS-KO (C), and hnRNPA1-KO (D) cells. Dashed lines indicate where the original blot image was spliced to juxtapose lanes that were non-contiguous. HSP70 or HSP90 served as a loading control.

(E) RT-qPCR analysis of *STMN2* mRNA in WT and RBP-KO cell lines. Data are means ± SEM from three biological replicates. $*p < 0.05$, $**p < 0.01$; N.S., not significant (one-way ANOVA followed by Tukey's post hoc test).

(F–H) Immunoblot analysis of RBPs in WT cells and in MATR3-KO (F), FUS-KO (G), and hnRNPA1-KO (H) cells complemented with a corresponding doxycycline-inducible RBP vector and exposed (or not) to doxycycline (DOX). Dashed line indicates where the original blot image was spliced to juxtapose lanes that were non-contiguous. HSP70 or HSP90 served as a loading control.

Appendix Figure S2

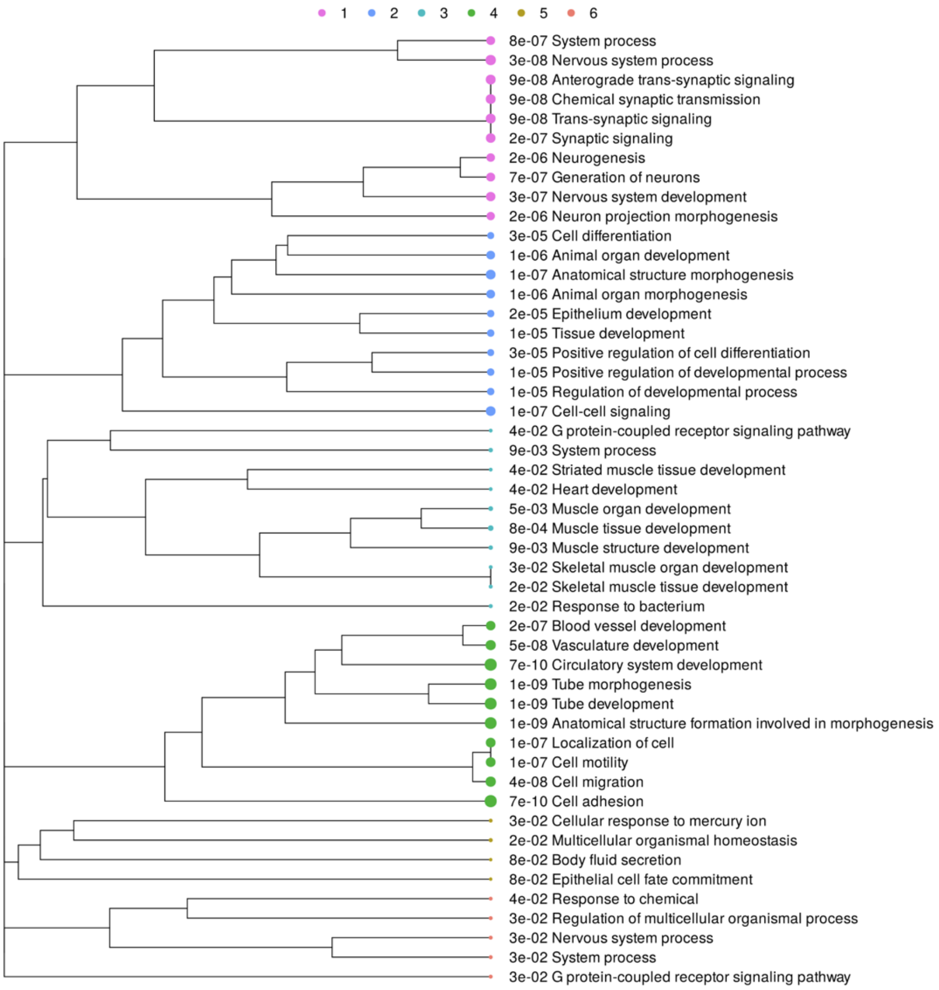

Appendix Figure S2. GO analysis of RNA-seq data from RBP-KO cells, related to Figure 1

Genes in clusters 1 to 6 shown in Fig. 1B were subjected to GO biological process analysis. The numbers indicate the FDR values of each GO term, and the circle size indicates fold enrichment.

Appendix Figure S3

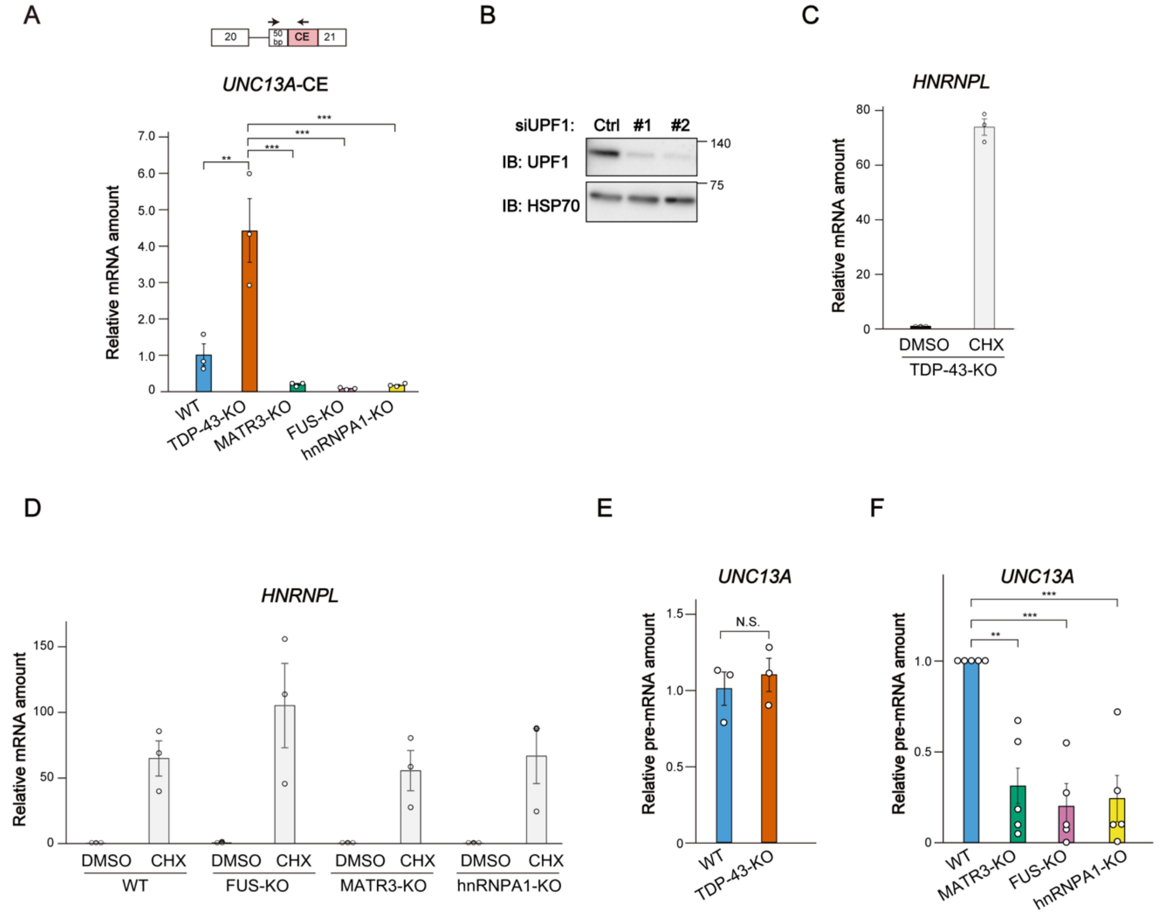

Appendix Figure S3. Inhibition of NMD and detection of *UNC13A* pre-mRNA in RBP-KO cells, related to Figure 2

(A) RT-qPCR analysis of *UNC13A* transcripts including the cryptic exon (CE) in WT cells and the four RBP-KO cell lines. The PCR primer sequences for CE detection were referenced from Koike *et al.* (2023) and their locations are illustrated at the top. Data are means ± SEM from three biological replicates. $^{**}p < 0.01$, $^{***}p < 0.001$ (one-way ANOVA followed by Tukey's post hoc test).

(B) Immunoblot analysis of UPF1 in TDP-43-KO cells transfected with a GC duplex (negative control) or either of two siRNAs targeting *UPF1*. HSP70 served as a loading control.

(C and D) RT-qPCR analysis of *HNRNPL* mRNA (positive control sensitive to NMD) in TDP-43-KO cells (C) or in WT, MATR3-KO, FUS-KO, and hnRNPA1-KO cells (D) after treatment with cycloheximide or DMSO vehicle. Data are means ± SEM from three biological replicates.

(E) RT-qPCR analysis of *UNC13A* pre-mRNA in WT and TDP-43-KO cells. Data are means ± SEM from three biological replicates. N.S. (Student's *t* test).

(F) RT-qPCR analysis of *UNC13A* pre-mRNA in WT, MATR3-KO, FUS-KO, and hnRNPA1-KO cells. Data are means ± SEM from five biological replicates. $^{**}p < 0.01$, $^{***}p < 0.001$ (one-way ANOVA followed by Tukey's post hoc test).

Appendix Figure S4

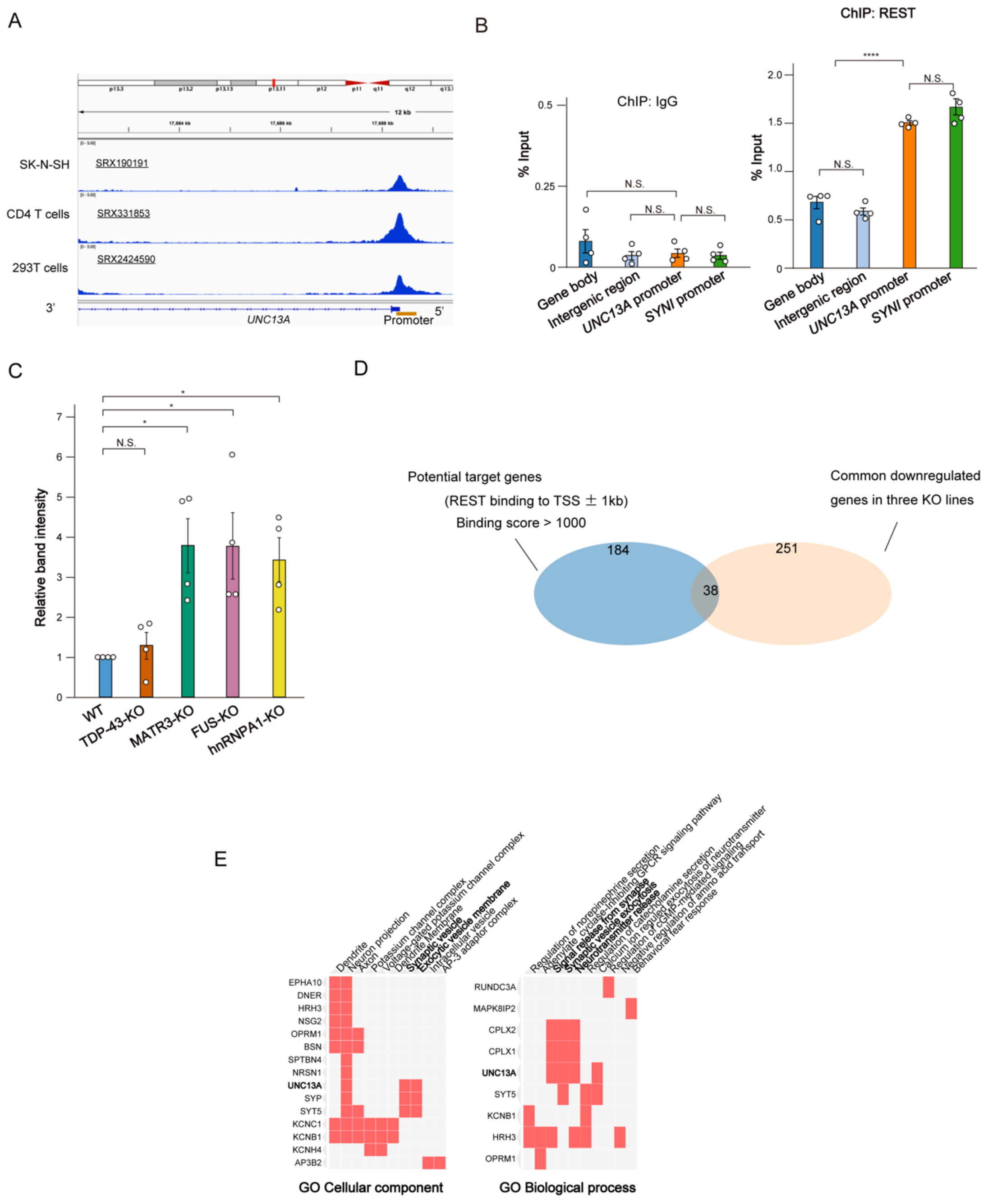

Appendix Figure S4. REST binds to the *UNC13A* promoter and suppresses the expression of various genes in RBP-KO cells, related to Figure 3

(A) Alignment of ChIP-seq reads for REST to the *UNC13A* promoter with the use of ENCODE data sets. ChIP-seq data from SK-N-SH cells, HEK293T cells, and CD4 T cells

show specific binding of REST to the *UNC13A* promoter region (chr19:17688234-17688572 in Hg38).

(B) ChIP-qPCR analysis of REST and IgG (negative control) binding to the *UNC13A* promoter in SH-SY5Y cells. The gene body of *UNC13A* and an intergenic region were examined as negative controls to which REST does not bind. The promoter region *Synapsin1* (*SYN1)*, which is known to bind REST, was examined as a positive control. Data are means from four biological replicates. ****p < 0.0001, N.S. (one-way ANOVA followed by Tukey's post hoc test).

(C) Quantification of the band intensity for REST (CST antibody preparation) normalized by that for HSP70 in immunoblots similar to that shown in Fig. 3F. Data are means ± SEM for four biological replicates. *p < 0.05, N.S (one-way ANOVA followed by Tukey's post hoc test).

(D) Venn diagram showing the overlap between potential target genes of REST and commonly downregulated genes in MATR3-KO, FUS-KO, and hnRNPA1-KO cells. Potential target genes, defined as those to which REST binds within ±1 kb of the TSS, were identified by cross-referencing ENCODE ChIP-seq data with ChIP-Atlas. Genes with a binding score as calculated with MACS2 and STRING of >1000 were considered potential target genes.

(E) GO analysis of the 38 potential REST target genes identified among the commonly downregulated genes in the three RBP-KO cell lines as in (D). These genes were annotated for GO terms with the Enrichr tool (https://maayanlab.cloud/Enrichr).

Appendix Figure S5

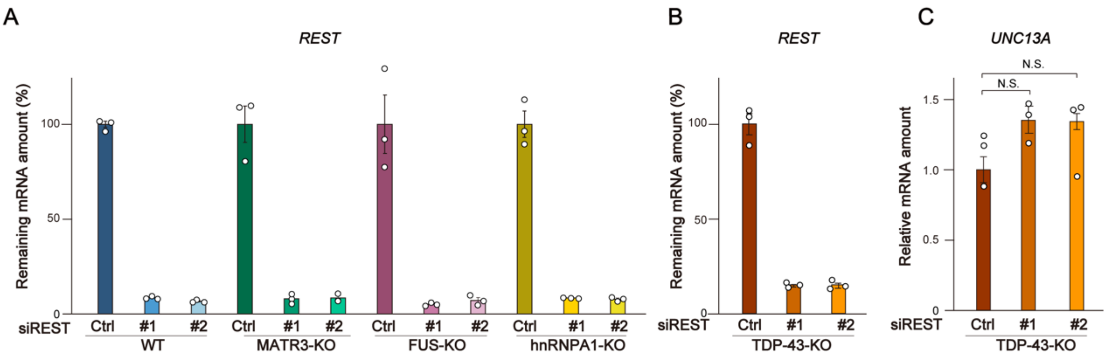

Appendix Figure S5. Knockdown of REST in RBP-KO cells, related to Figure 4

(A) RT-qPCR analysis of *REST* mRNA in WT, MATR3-KO, FUS-KO, and hnRNPA1-KO cell lines transfected with either a GC duplex (negative control) or one of two different REST siRNAs. Data are means ± SEM from three biological replicates.

(B and C) RT-qPCR analysis of *REST* (B) and *UNC13A* (C) mRNAs in TDP-43-KO cells transfected as in (A). Data are means ± SEM from three biological replicates. N.S. (one-way ANOVA followed by Tukey's post hoc test.)

Appendix Figure S6

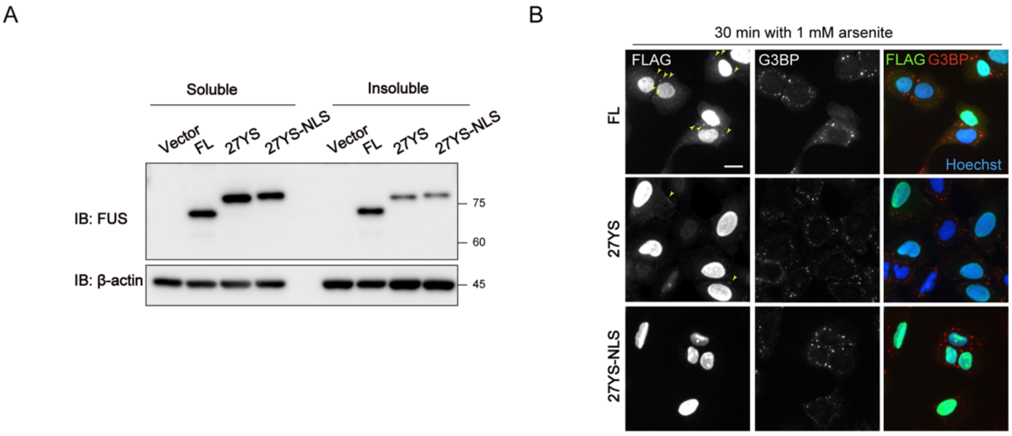

Appendix Figure S6. Characterization of FUS 27YS and 27YS-NLS mutants, related to Figure 6

(A) Immunoblot analysis of FUS in FUS-KO cells expressing FL, 27YS, or 27YS-NLS forms of FUS. The cell lysates were prepared in the presence of 0.5% Nonidet P-40 detergent and centrifuged at 20,000 × *g* for 15 min at 4°C, and the resulting supernatant was collected as the soluble fraction. The pellet was subjected to ultrasonic treatment and dissolved in urea buffer to obtain the insoluble fraction. β-actin served as a loading control.

(B) Immunostaining of U2OS cells expressing FL, 27YS, or 27YS-NLS forms of FUS after treatment with sodium arsenite (1 mM, 30 min). Cells were stained for FLAG, the stress granule (SG) marker G3BP, and Hoechst. The yellow arrowheads indicate stress-induced cytoplasmic FUS granules. Scale bar = 20 µm.

Appendix Figure S7

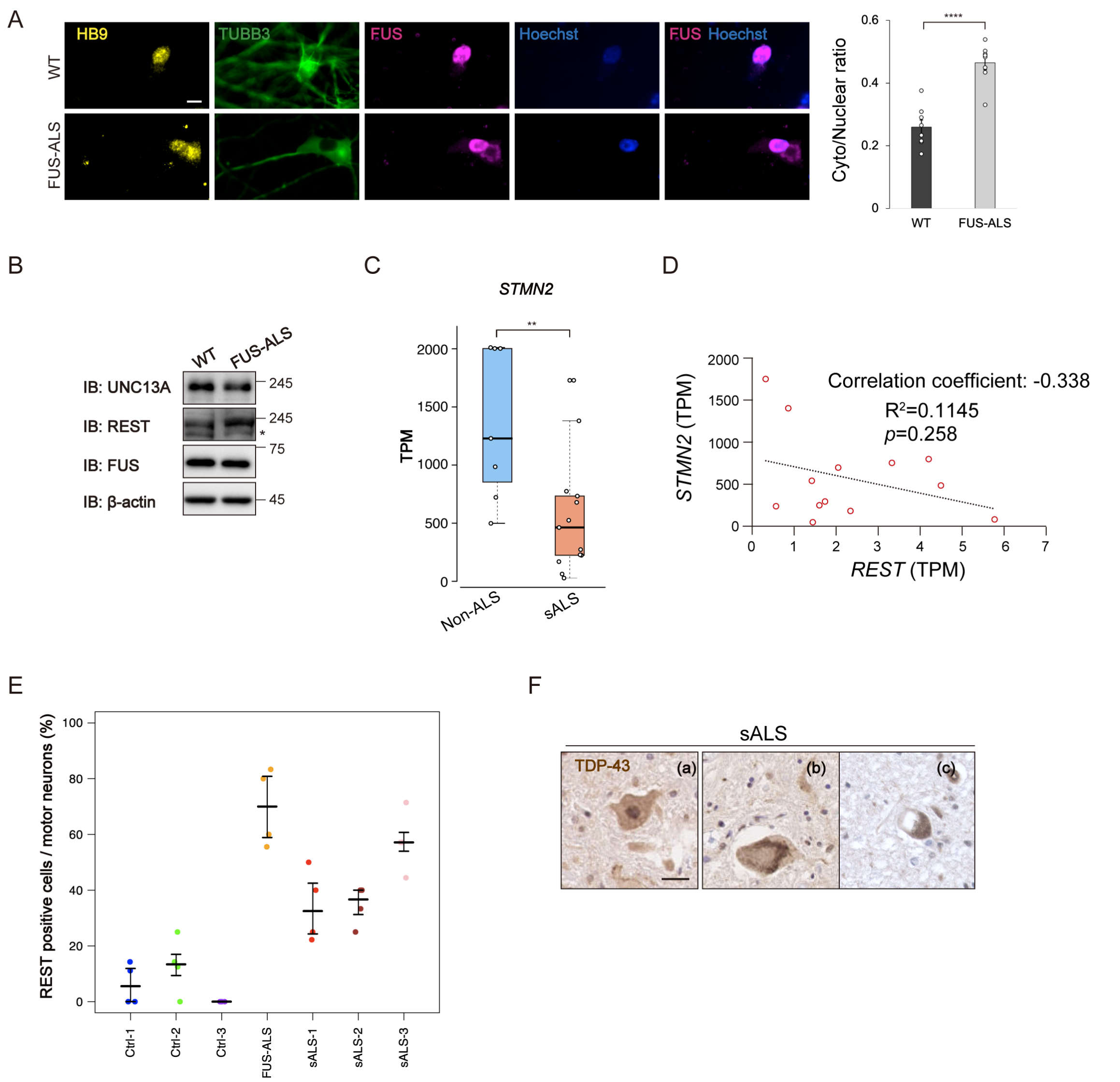

Appendix Figure S7. Overexpression of REST in FUS-ALS iMNs and individuals with familial or sporadic ALS, related to Figure 7

(A) (Left) Immunostaining of WT and FUS P525L/+ (FUS-ALS) iMNs. Cells were stained with HB9 (a motor neuron marker), Tubulin βIII (TUBB3), FUS, and Hoechst. Merged images of FUS and Hoechst are also shown. Scale bar = 10 µm. (Right) Comparison of FUS cytoplasmic-to-nuclear (Cyto/Nuclear) intensity ratios between WT and FUS-ALS iMNs (n = 8 per group). Nuclear and cytoplasmic FUS signals were quantified using ImageJ. Data are means ± SEM. ****$p < 0.0001$ (Student's *t* test).

(B) Immunoblot of WT and FUS-ALS iMNs. Asterisk indicates nonspecific bands. β-actin served as a loading control.

(C) Comparison of *STMN2* expression in lumbar motor neurons between control (non-ALS) individuals and individuals with sporadic ALS (sALS) in the GSE76220 data set. **$p < 0.01$ (Mann-Whitney U test).
(D) Scatter plot showing the relation between the expression levels of *REST* and *STMN2* in lumbar motor neurons for sALS patients in the GSE76220 data set. Each red dot represents one sample. The dashed line indicates the linear regression fit. The R^2 value shows the proportion of variance in *STMN2* expression explained by *REST* expression, and the p value was calculated with Pearson's correlation test.
(E) Quantification of REST-positive cells among anterior horn neurons for three control individuals, an individual with familial ALS associated with a *FUS* mutation (R521C/+), and three sALS patients shown in Fig. 7H, J. The percentage of REST-positive cells was quantified per square millimeter in four sections per sample. Both individual data points (four per sample) and their average are presented. Horizontal bars indicate the median (longer line) and interquartile range (shorter lines at the first and third quartiles, Q1 and Q3).
(F) Immunohistochemical staining of TDP-43 in spinal motor neurons of three sALS patients. TDP-43 pathology was apparent in patients (b) and (c), but not in patient (a). Scale bar, 25 µm.

**Appendix Table S1: ALS-associated genes categorized in ALSoD**			
**Definitive ALS genes**	**Strong evidence**	**Moderate evidence**	**Clinical modifier**
ANXA11	ATXN1	ANG	ATXN2
C9orf72	CCNF	ARHGEF28	CAMTA1
CHCHD10	CFAP410	CDH22	ENAH
EPHA4	HFE	CHMP2B	
FUS	NIPA1	CNTN6	
HNRNPA1	SCFD1	CRYM	
KIF5A	TUBA4A	CSNK1G3	
NEK1		CX3CR1	
OPTN		DAO	
PFN1		DNAJC7	
SOD1		DNMT3A	
TARDBP		ERBB4	
TBK1		FIG4	
UBQLN2		GLE1	
UNC13A		GPX3	
VAPB		LMNB1	
VCP		SARM1	
		SMN1	
		SQSTM1	
		SS18L1	
		TNIP1	

**Appendix Table S2: ChIP-seq Datasets for TFs Binding to the *UNC13A* Promoter Region**		
**Dataset ID**	**Antigen**	**Cell line**
SRX100380	REST	SK-N-SH
SRX190191	REST	SK-N-SH
SRX4679525	REST	SK-N-MM
SRX100414	REST	PFSK-1
SRX190242	REST	PFSK-1
SRX100417	REST	U-87 MG
SRX4679528	REST	CHLA-90
SRX11051031	JUN	hTERT RPE-1
SRX11051032	JUN	hTERT RPE-1
SRX11051045	JUN	hTERT RPE-1
SRX11051046	JUN	hTERT RPE-1
SRX100543	YY1	SK-N-SH
SRX190353	YY1	SK-N-SH
SRX8901838	YY1	GSC23
SRX190194	GATA3	SK-N-SH
SRX190237	SIN3A	PFSK-1

**Appendix Table S3: Transcription Factor Binding Motif Analysis in Promoter Regions of Commonly Downregulated Genes**			
**Motif**	**TF**	**TF family**	**FDR**
GGGG	ZNF202	C2H2 ZF	2.00E-07
GGGGCCCAAGGGGG	PLAG1	C2H2 ZF	3.60E-05
CG	CGBP	CxxC	2.00E-04
GGGGGTGG	ZNF281	C2H2 ZF	3.40E-04
CCCCGGGC	TFAP2A	AP-2	5.10E-04
CCG	DNMT1	CxxC	6.10E-04
CCCCGGGC	TFAP2C	AP-2	6.10E-04
GGGGGGT	ZIC5	C2H2 ZF	6.10E-04
TGCGGG	GCM1	GCM	6.70E-04
GGGGATTCCC	NFKB1	Rel	1.00E-03
GGCGCTGTCCGTGGTGCTGAA	REST	C2H2 ZF	1.00E-03
GGGGGGGGGTGGTTTGGGG	RREB1	C2H2 ZF	2.40E-03
GCGGGGGCGGGG	EGR1	C2H2 ZF	3.70E-03
GAGGGGGAA	MZF1	C2H2 ZF	3.70E-03
TGCGGG	ZBTB1	C2H2 ZF	4.10E-03
CG	MLL	CxxC	4.10E-03
GGGGGCGGGGC	SP2	C2H2 ZF	4.60E-03
GGAGGAGGAGGGGGAGGAGG	ZNF263	C2H2 ZF	5.80E-03
GGGGGGGGGCC	PATZ1	C2H2 ZF	6.20E-03
TTCAGCACCATGGACAGCGCC	REST	C2H2 ZF	6.20E-03
