# Supplementary figures and images for: ALS-associated RNA-binding proteins promote UNC13A transcription through REST downregulation
Source: EMBO J. 2025 Jul 24;44(17):4745–71. doi: 10.1038/s44318-025-00506-0 (PMC12402202; doi:10.1038/s44318-025-00506-0)

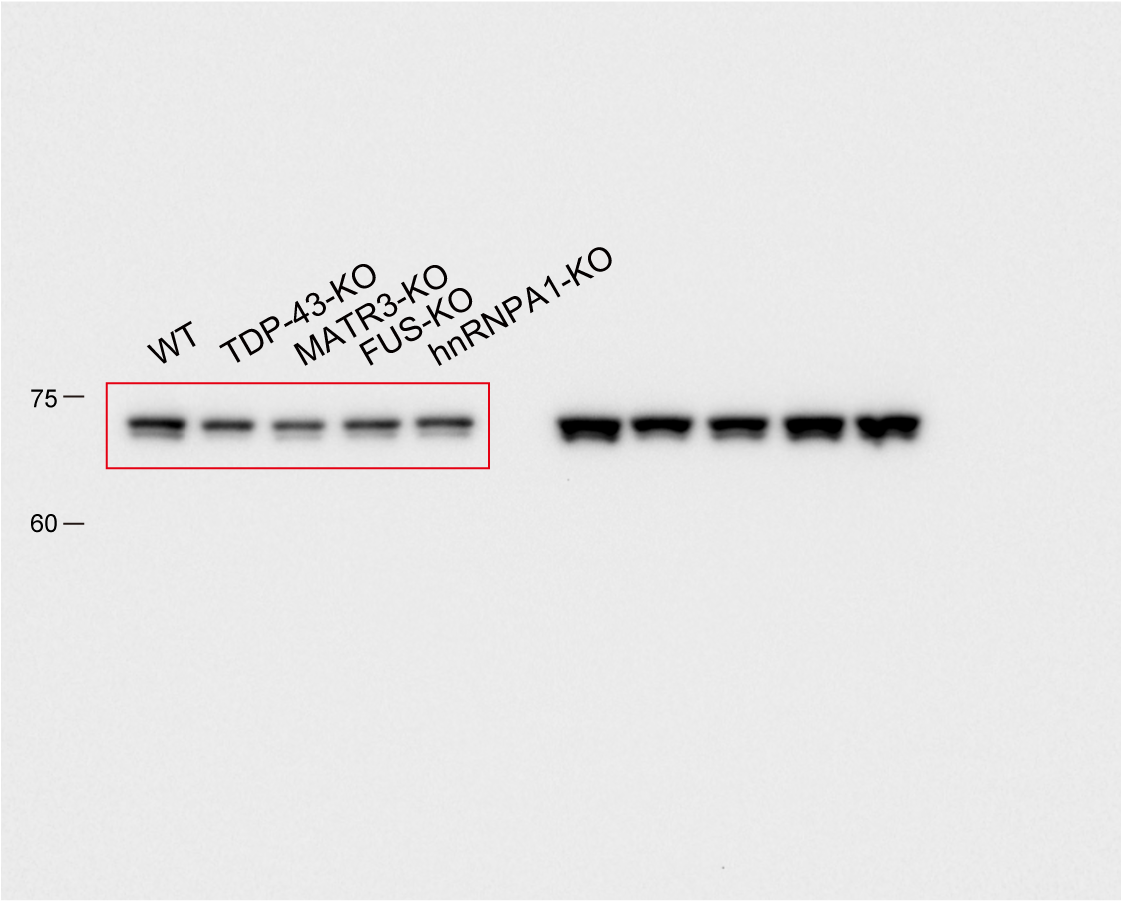

Supplement: Supplementary file 6 — Source data Fig. 1 [file 44318_2025_506_MOESM6_ESM.zip › Figure 1/Fig1E/Western_HSP70.tif]

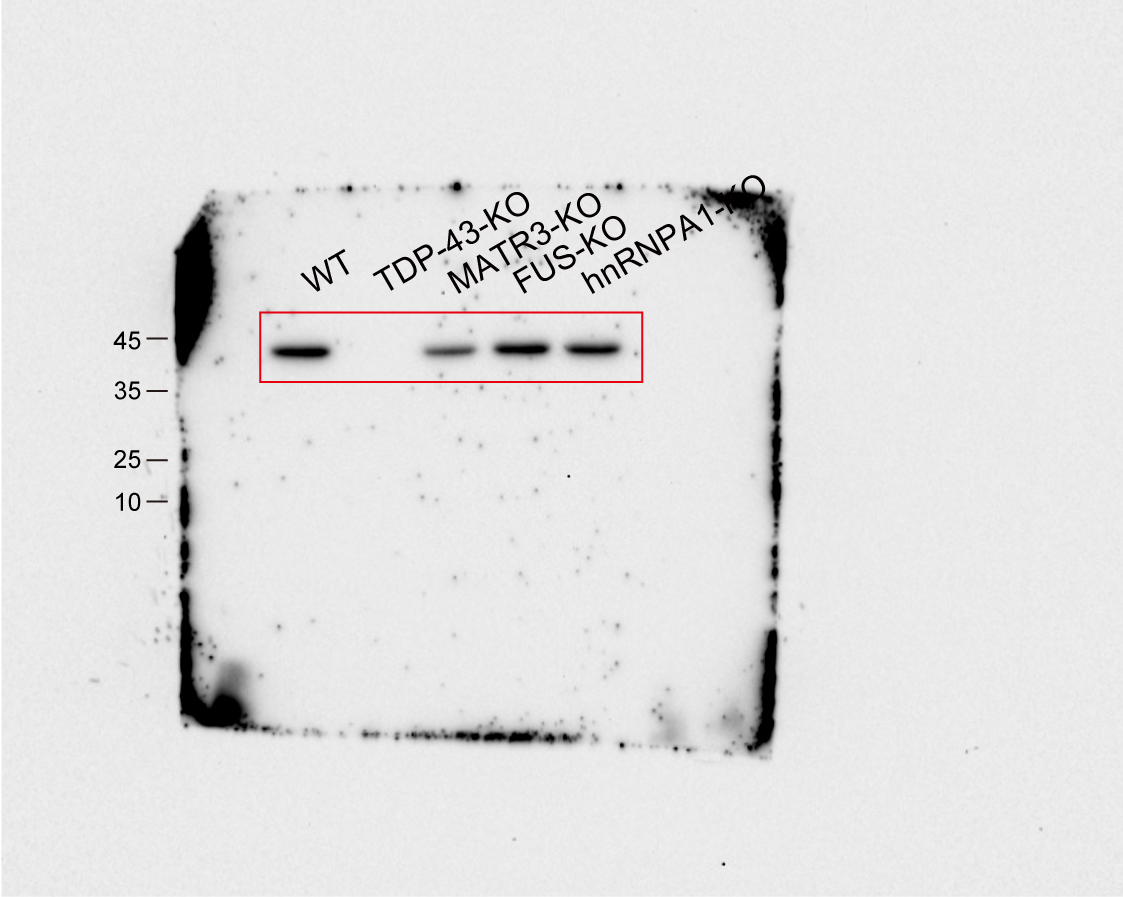

Supplement: Supplementary file 6 — Source data Fig. 1 [file 44318_2025_506_MOESM6_ESM.zip › Figure 1/Fig1E/Western_TDP-43.tif]

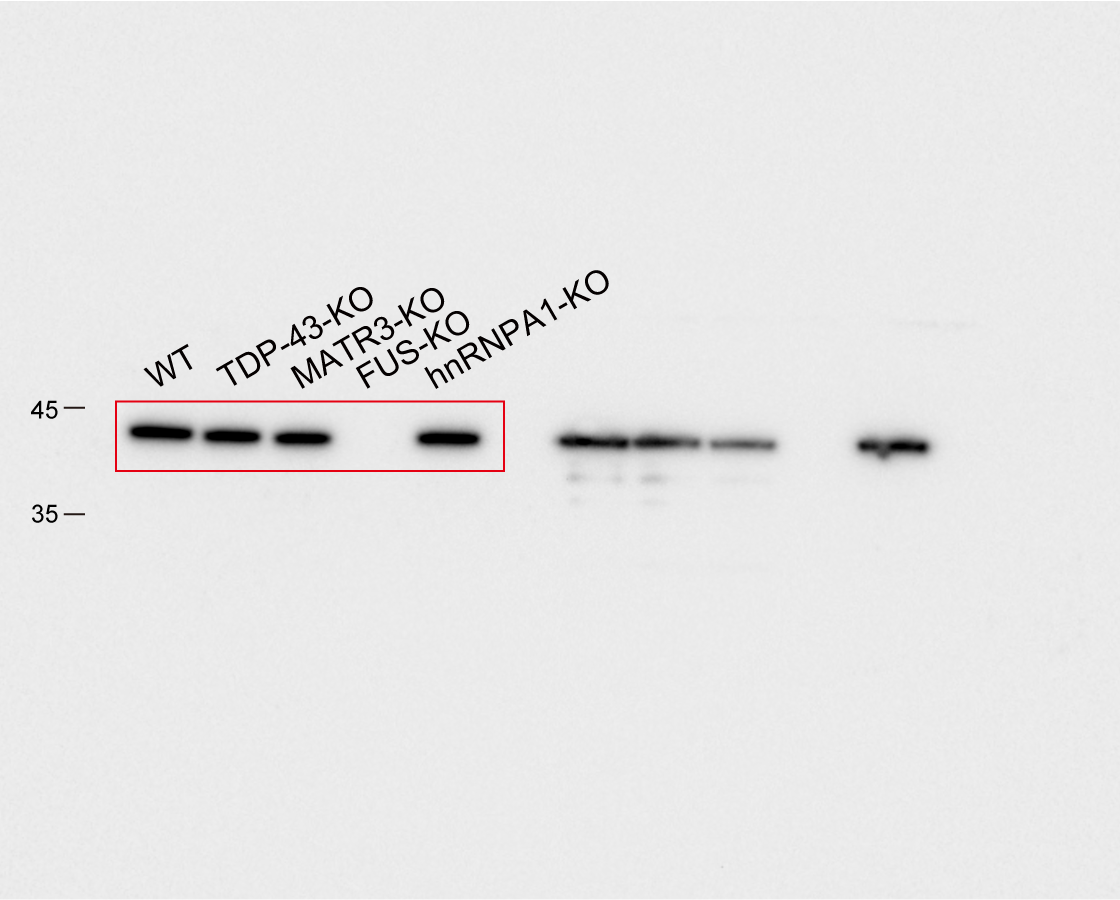

Supplement: Supplementary file 6 — Source data Fig. 1 [file 44318_2025_506_MOESM6_ESM.zip › Figure 1/Fig1E/Western_FUS.tif]

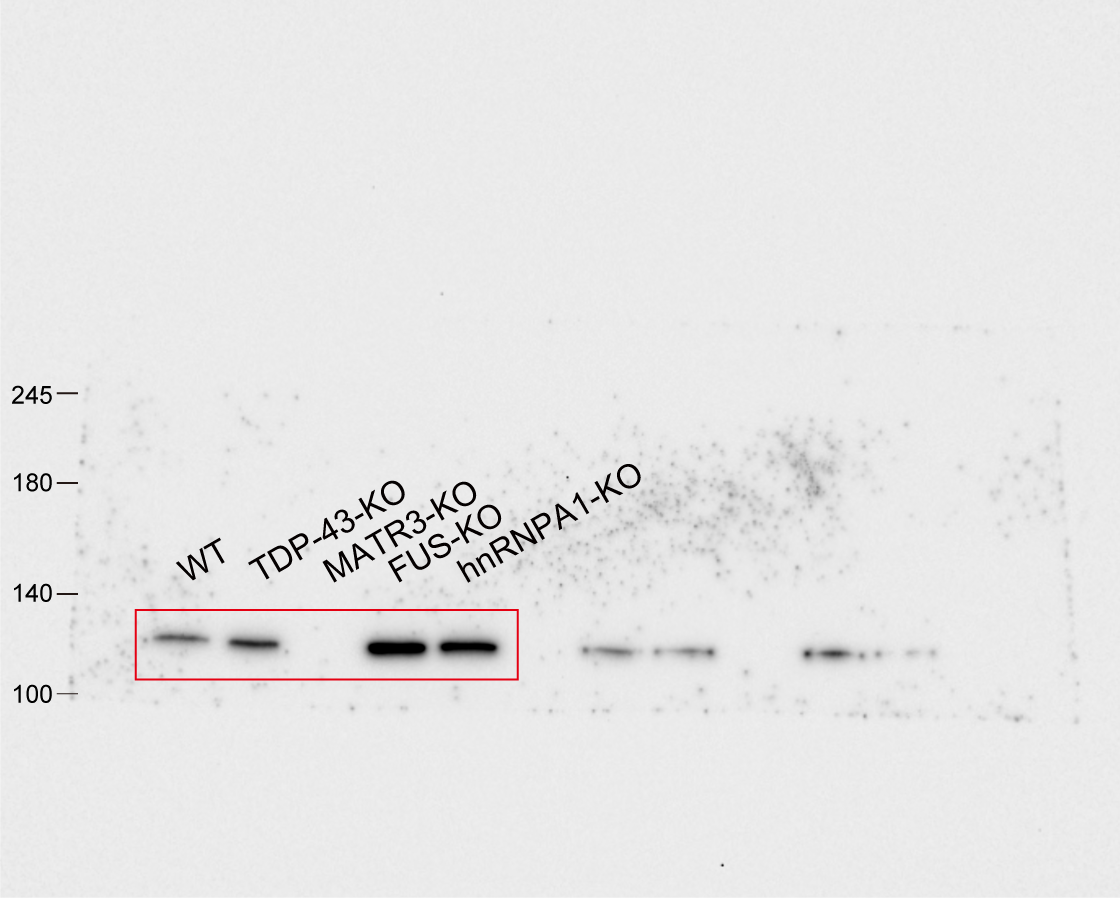

Supplement: Supplementary file 6 — Source data Fig. 1 [file 44318_2025_506_MOESM6_ESM.zip › Figure 1/Fig1E/Western_MATR3.tif]

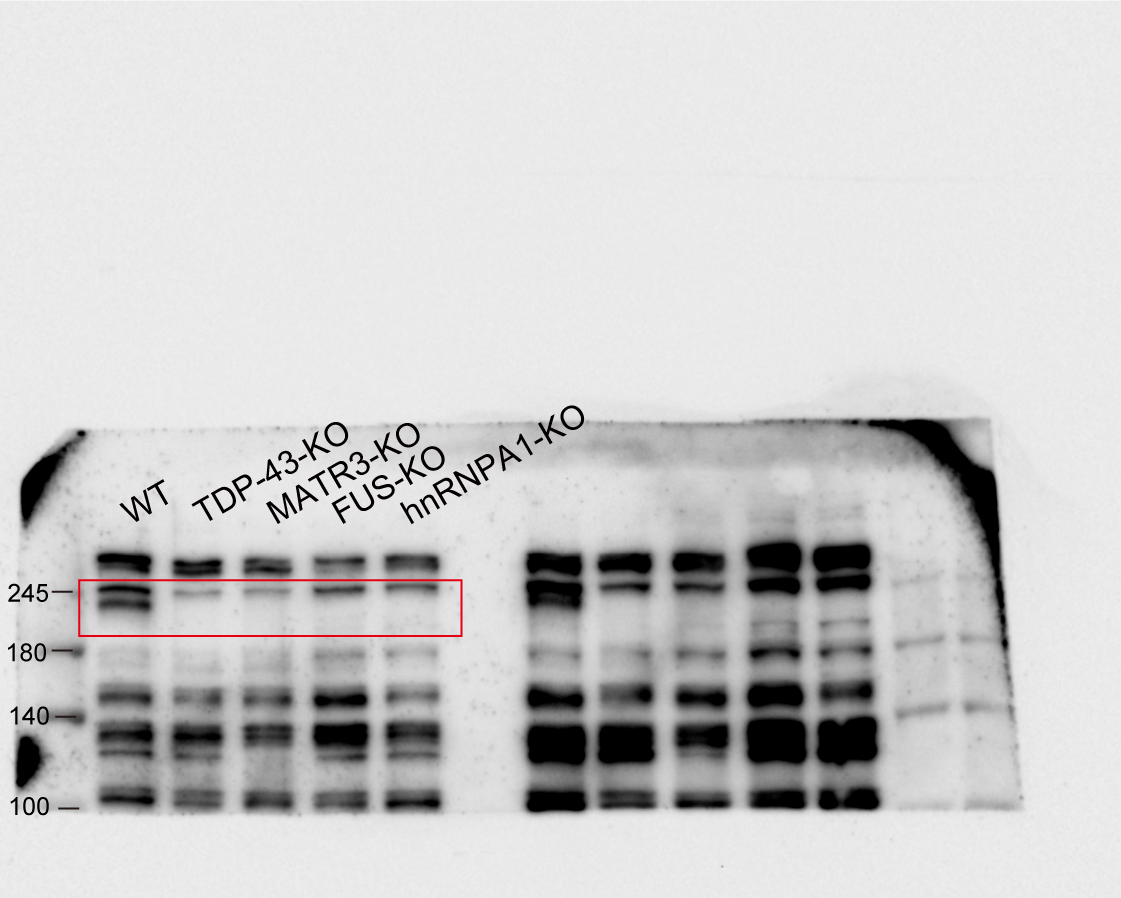

Supplement: Supplementary file 6 — Source data Fig. 1 [file 44318_2025_506_MOESM6_ESM.zip › Figure 1/Fig1E/Western_UNC13A.tif]

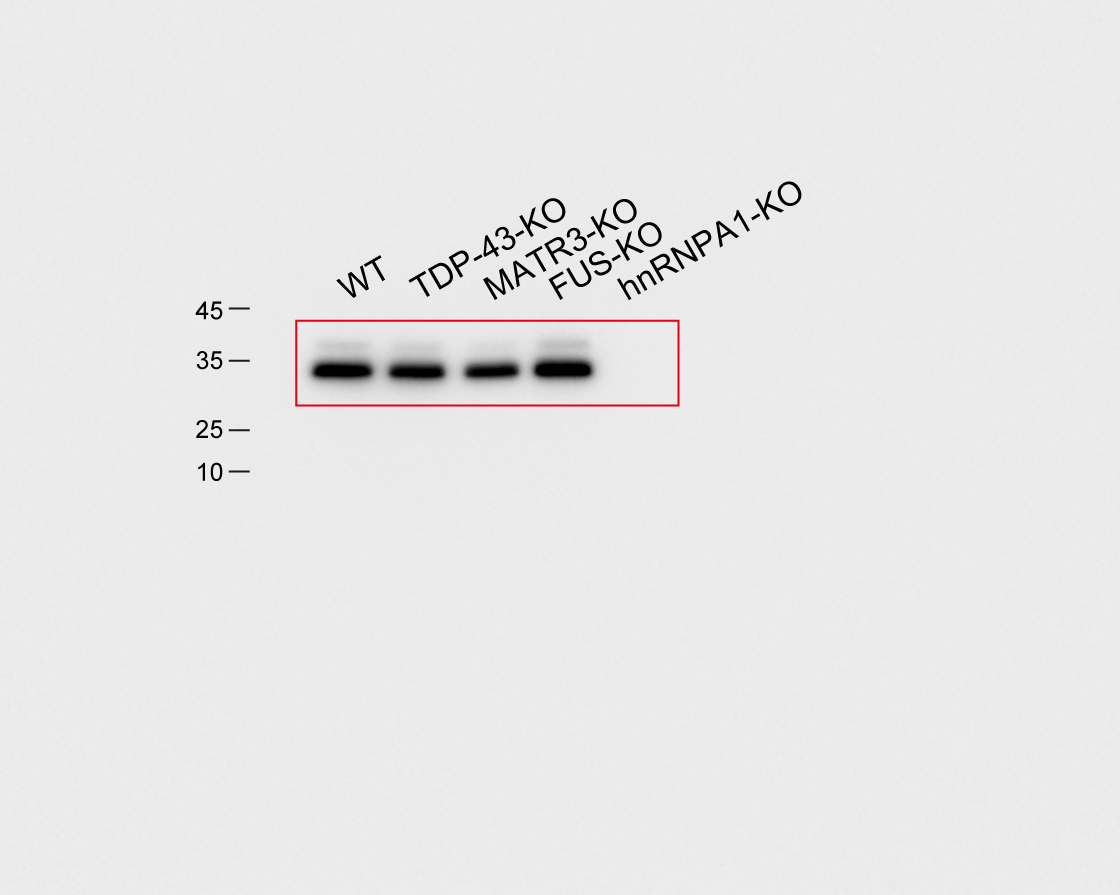

Supplement: Supplementary file 6 — Source data Fig. 1 [file 44318_2025_506_MOESM6_ESM.zip › Figure 1/Fig1E/Western_hnRNPA1.tif]

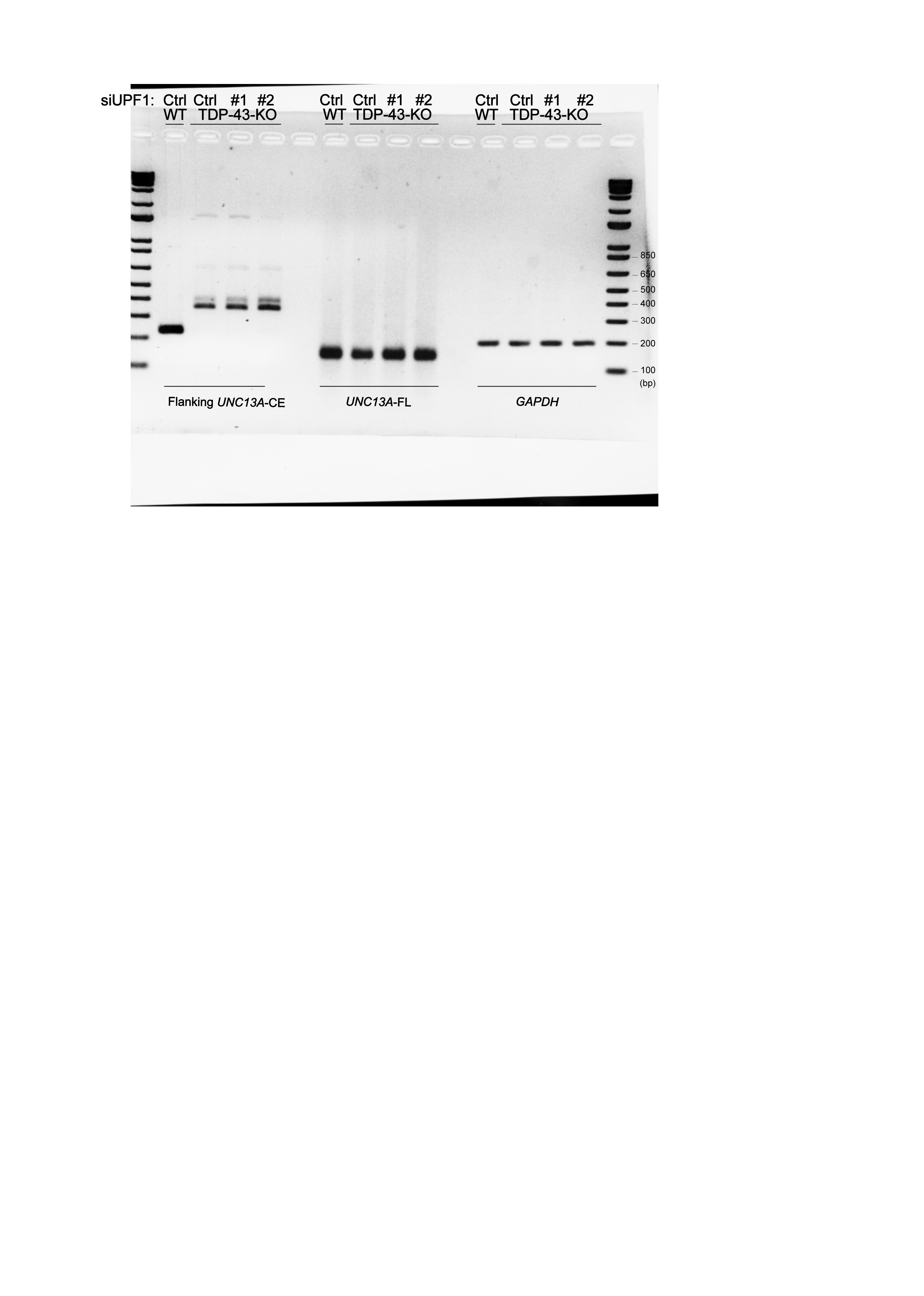

Supplement: Supplementary file 7 — Source data Fig. 2 [file 44318_2025_506_MOESM7_ESM.zip › Figure 2/Fig2B/AGE.tif]

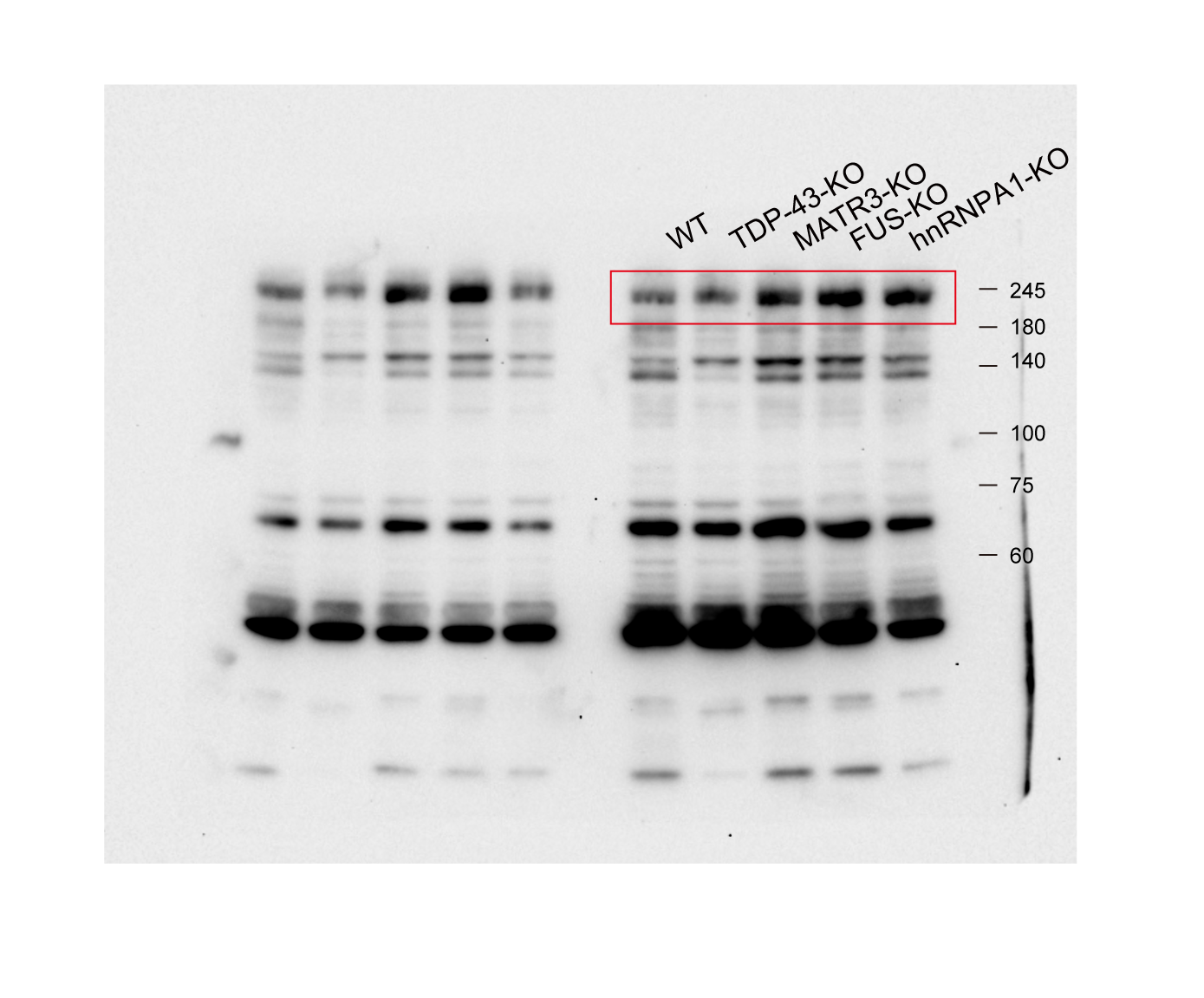

Supplement: Supplementary file 8 — Source data Fig. 3 [file 44318_2025_506_MOESM8_ESM.zip › Figure 3/Fig3F/Western_REST(Millipore).tif]

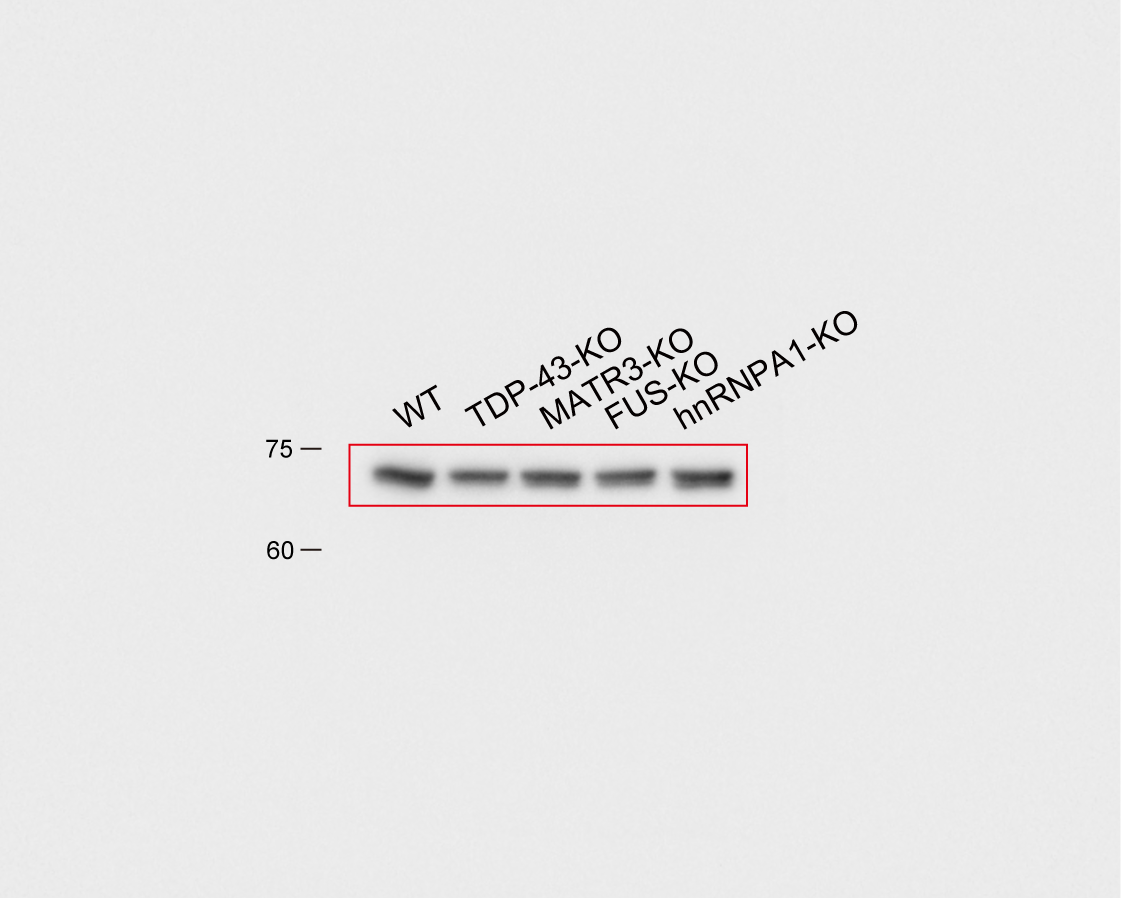

Supplement: Supplementary file 8 — Source data Fig. 3 [file 44318_2025_506_MOESM8_ESM.zip › Figure 3/Fig3F/Western_HSP70.tif]

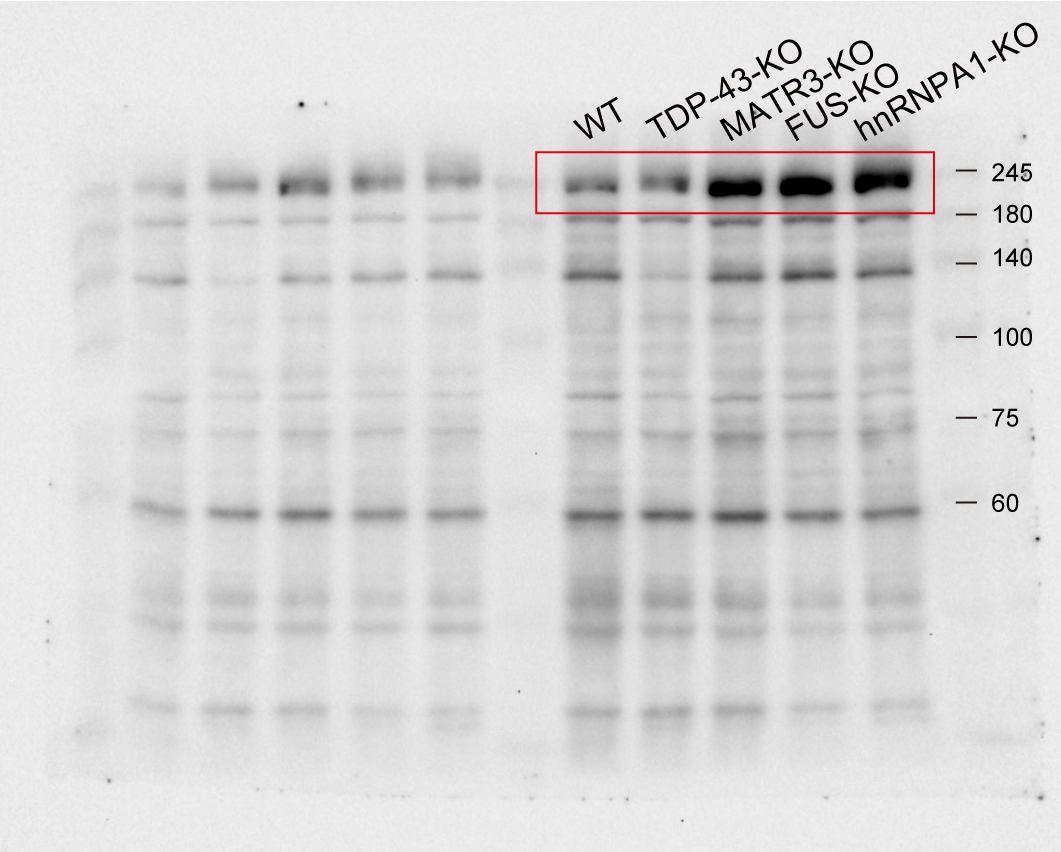

Supplement: Supplementary file 8 — Source data Fig. 3 [file 44318_2025_506_MOESM8_ESM.zip › Figure 3/Fig3F/Western_REST(CST).tif]

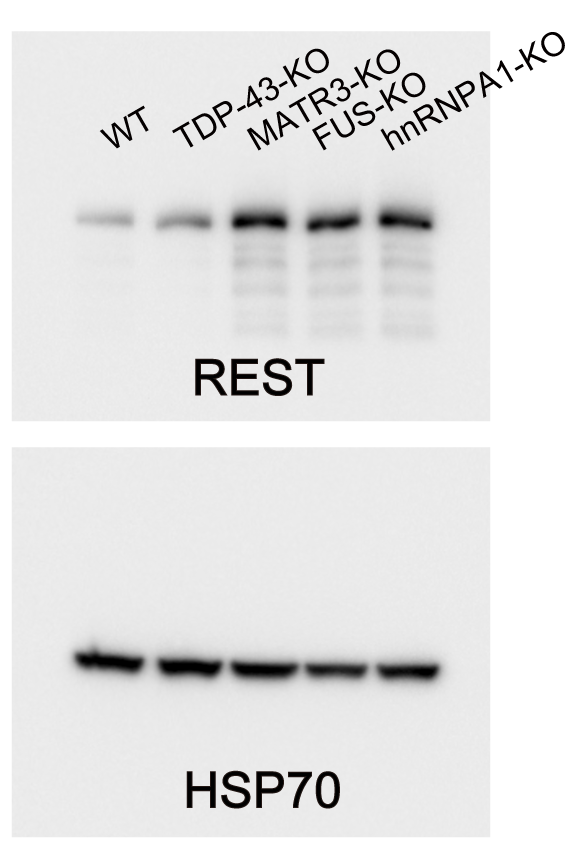

Supplement: Supplementary file 8 — Source data Fig. 3 [file 44318_2025_506_MOESM8_ESM.zip › Figure 3/Fig3F/Fig3F_replicates/replicate_4.tif]

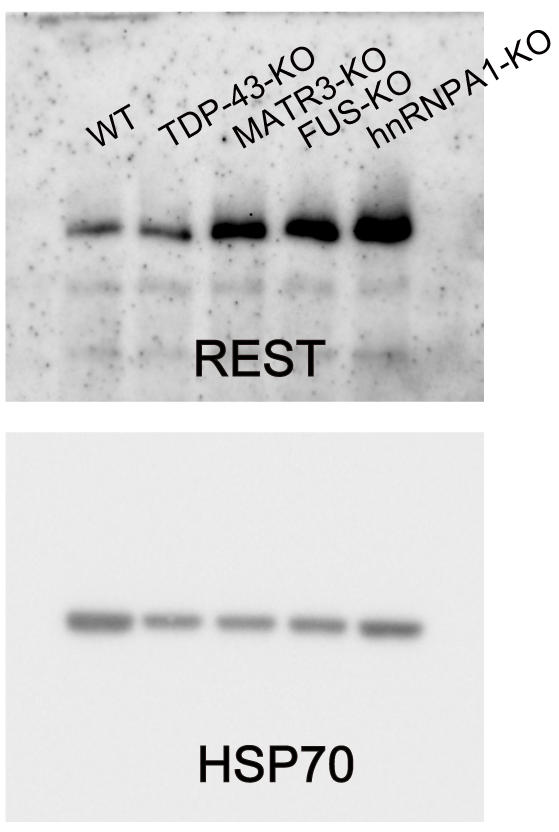

Supplement: Supplementary file 8 — Source data Fig. 3 [file 44318_2025_506_MOESM8_ESM.zip › Figure 3/Fig3F/Fig3F_replicates/replicate_3.tif]

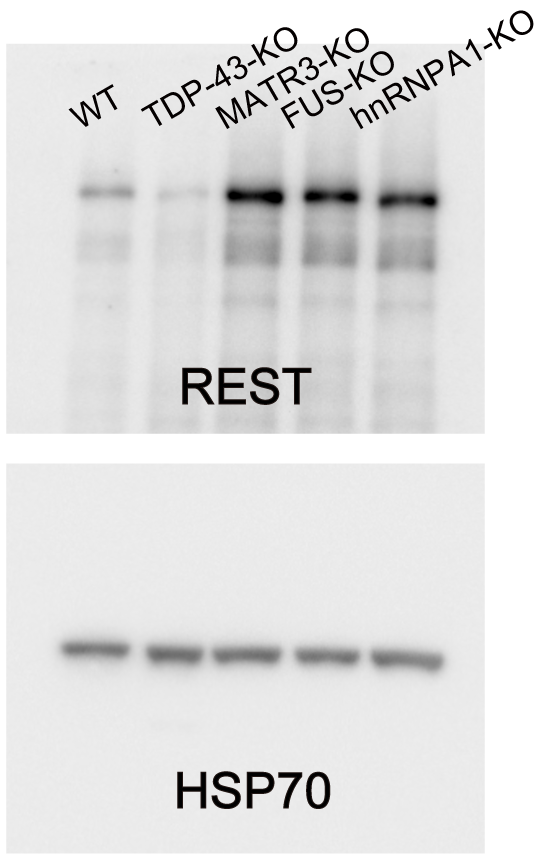

Supplement: Supplementary file 8 — Source data Fig. 3 [file 44318_2025_506_MOESM8_ESM.zip › Figure 3/Fig3F/Fig3F_replicates/replicate_2.tif]

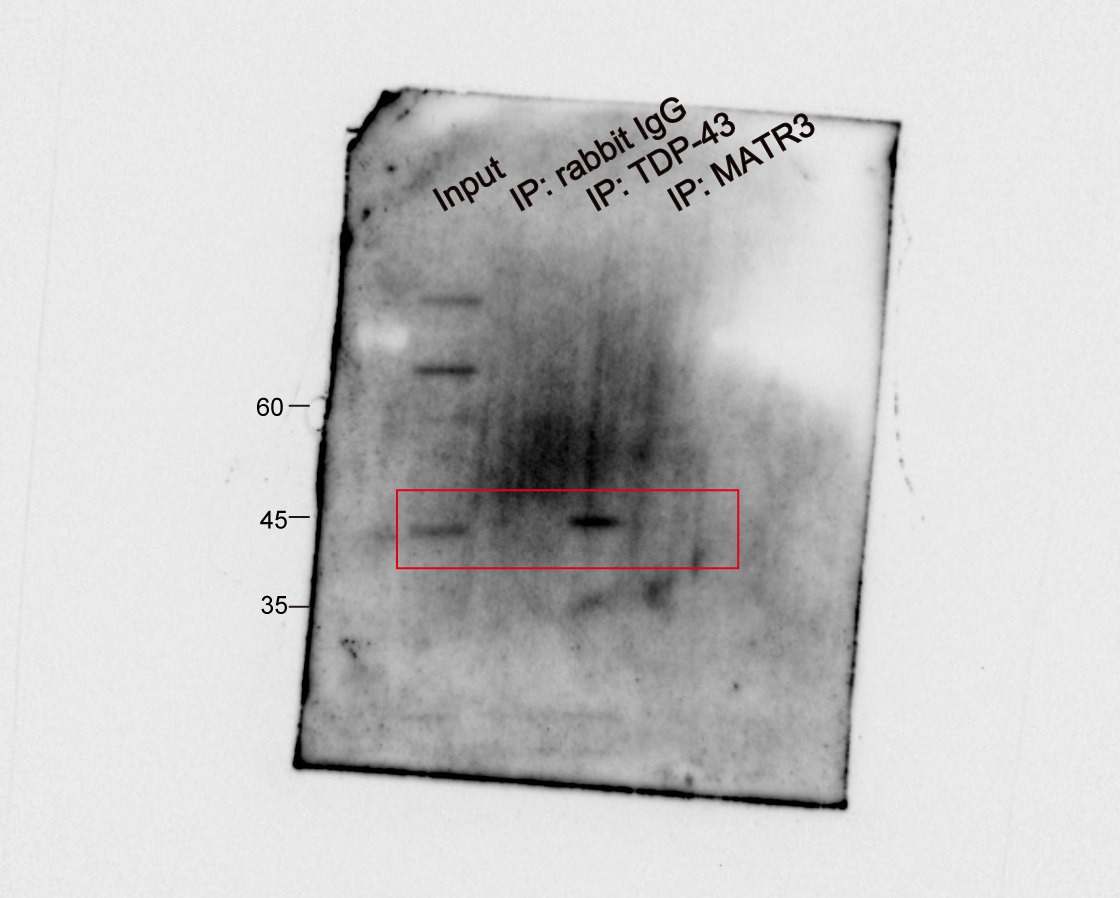

Supplement: Supplementary file 10 — Source data Fig. 5 [file 44318_2025_506_MOESM10_ESM.zip › Figure 5/Fig5B/EMBOJ_raw_Fig5B_TDP-43.tif]

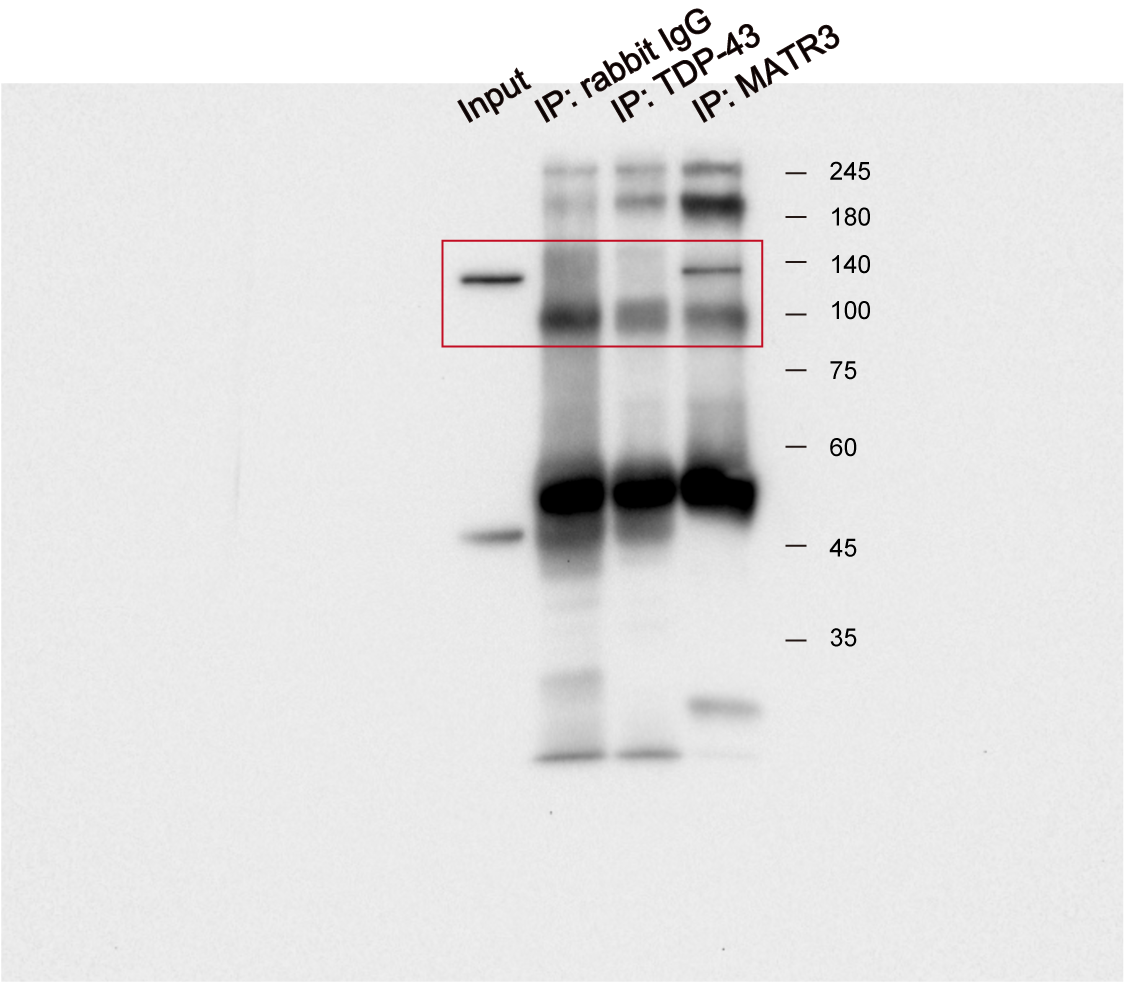

Supplement: Supplementary file 10 — Source data Fig. 5 [file 44318_2025_506_MOESM10_ESM.zip › Figure 5/Fig5B/EMBOJ_raw_Fig5B_MATR3.tif]

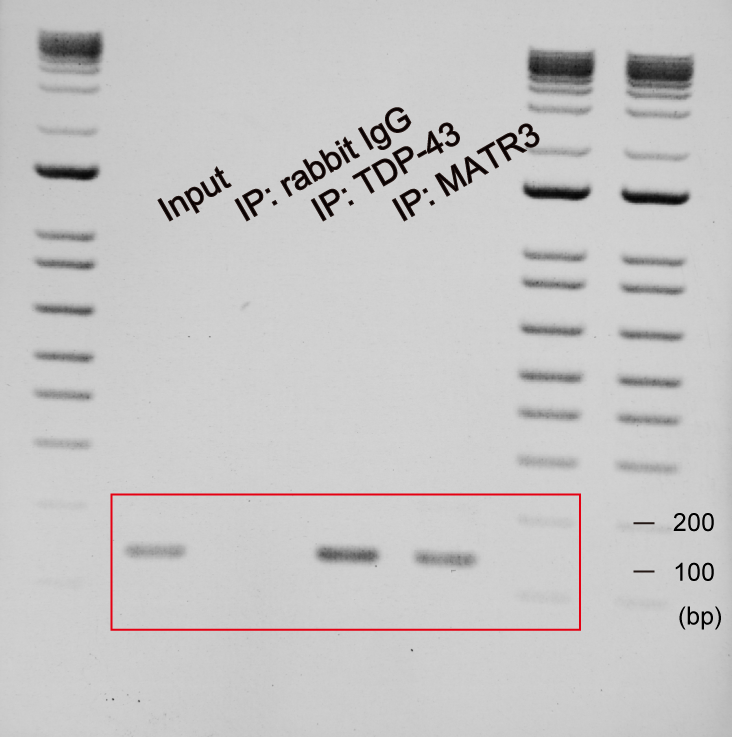

Supplement: Supplementary file 10 — Source data Fig. 5 [file 44318_2025_506_MOESM10_ESM.zip › Figure 5/Fig5E/EMBOJ_raw_Fig5E.tif]

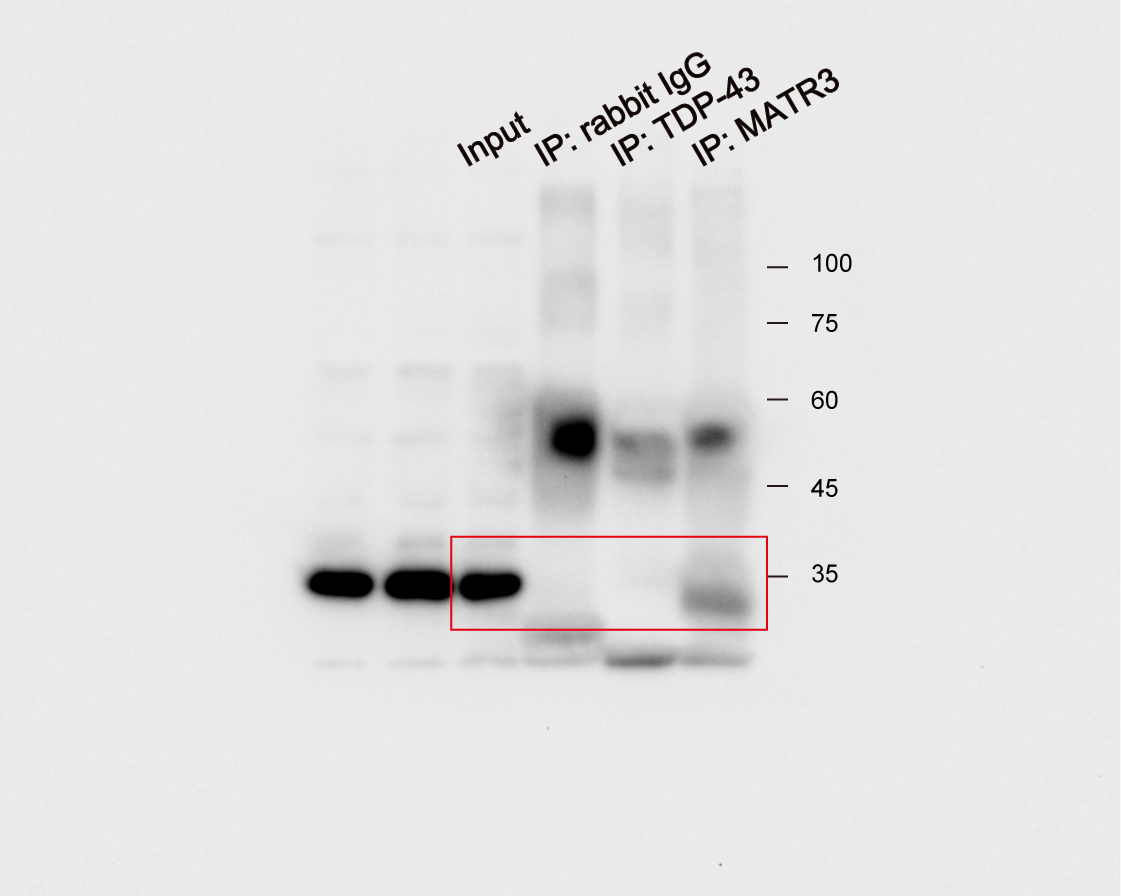

Supplement: Supplementary file 10 — Source data Fig. 5 [file 44318_2025_506_MOESM10_ESM.zip › Figure 5/Fig5D/EMBOJ_raw_Fig5D_hnRNPA1.tif]

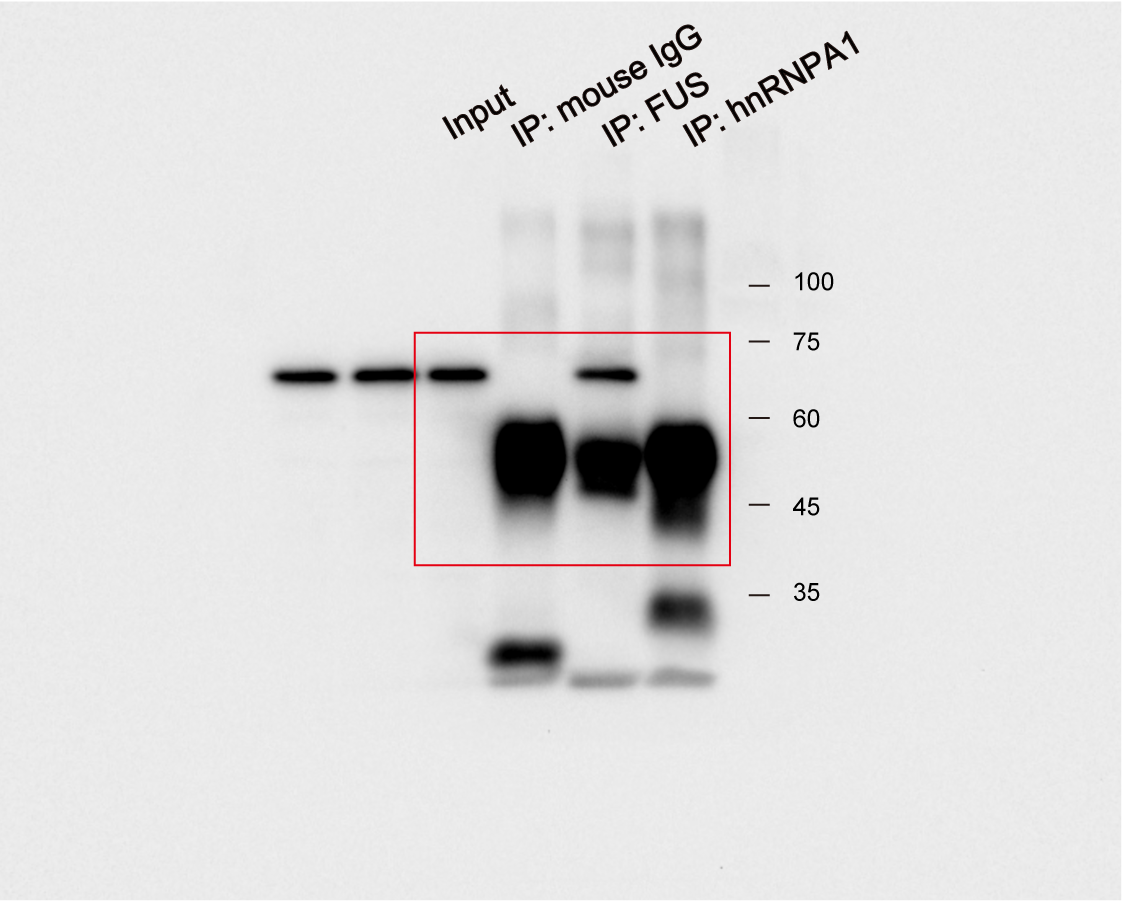

Supplement: Supplementary file 10 — Source data Fig. 5 [file 44318_2025_506_MOESM10_ESM.zip › Figure 5/Fig5D/EMBOJ_raw_Fig5D_FUS.tif]

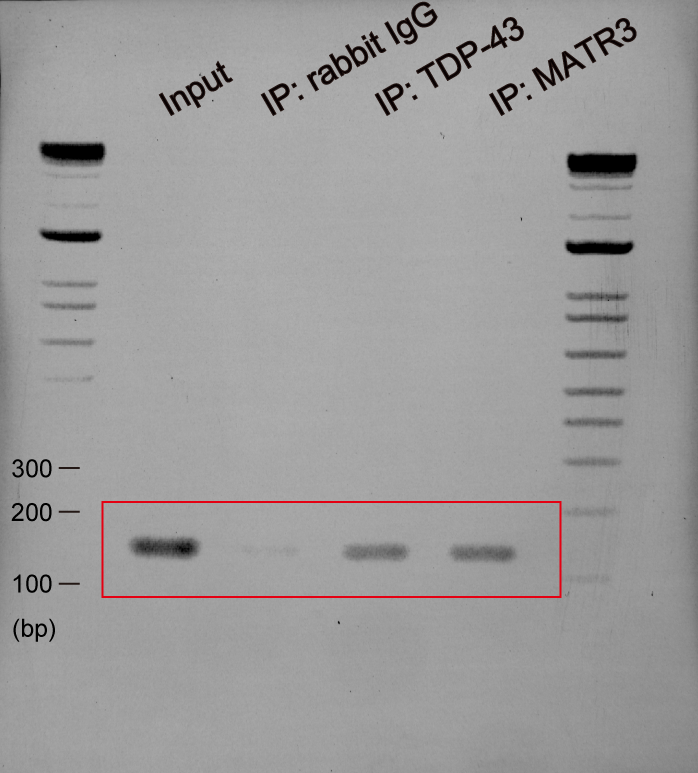

Supplement: Supplementary file 10 — Source data Fig. 5 [file 44318_2025_506_MOESM10_ESM.zip › Figure 5/Fig5C/EMBOJ_raw_Fig5C_TARDBP.tif]

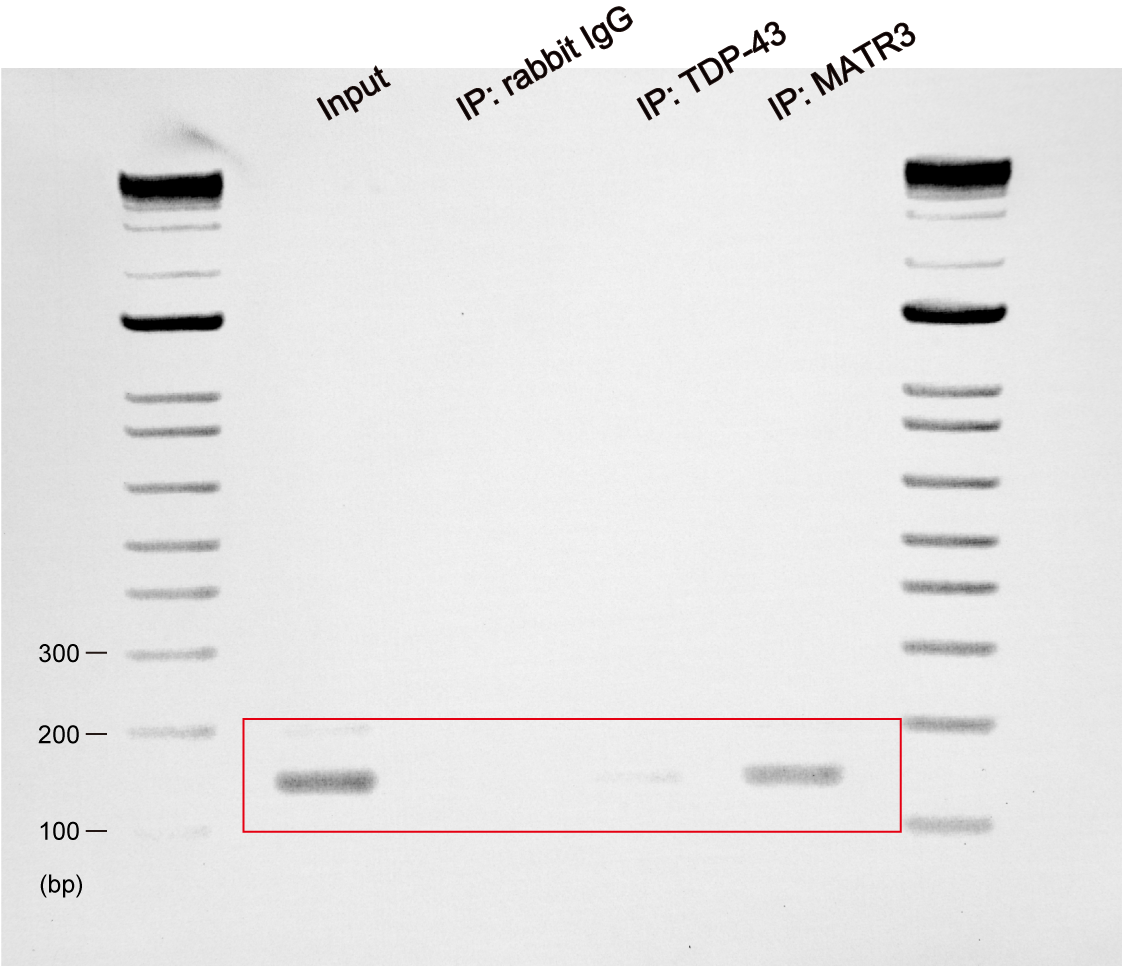

Supplement: Supplementary file 10 — Source data Fig. 5 [file 44318_2025_506_MOESM10_ESM.zip › Figure 5/Fig5C/EMBOJ_raw_Fig5C_REST.tif]

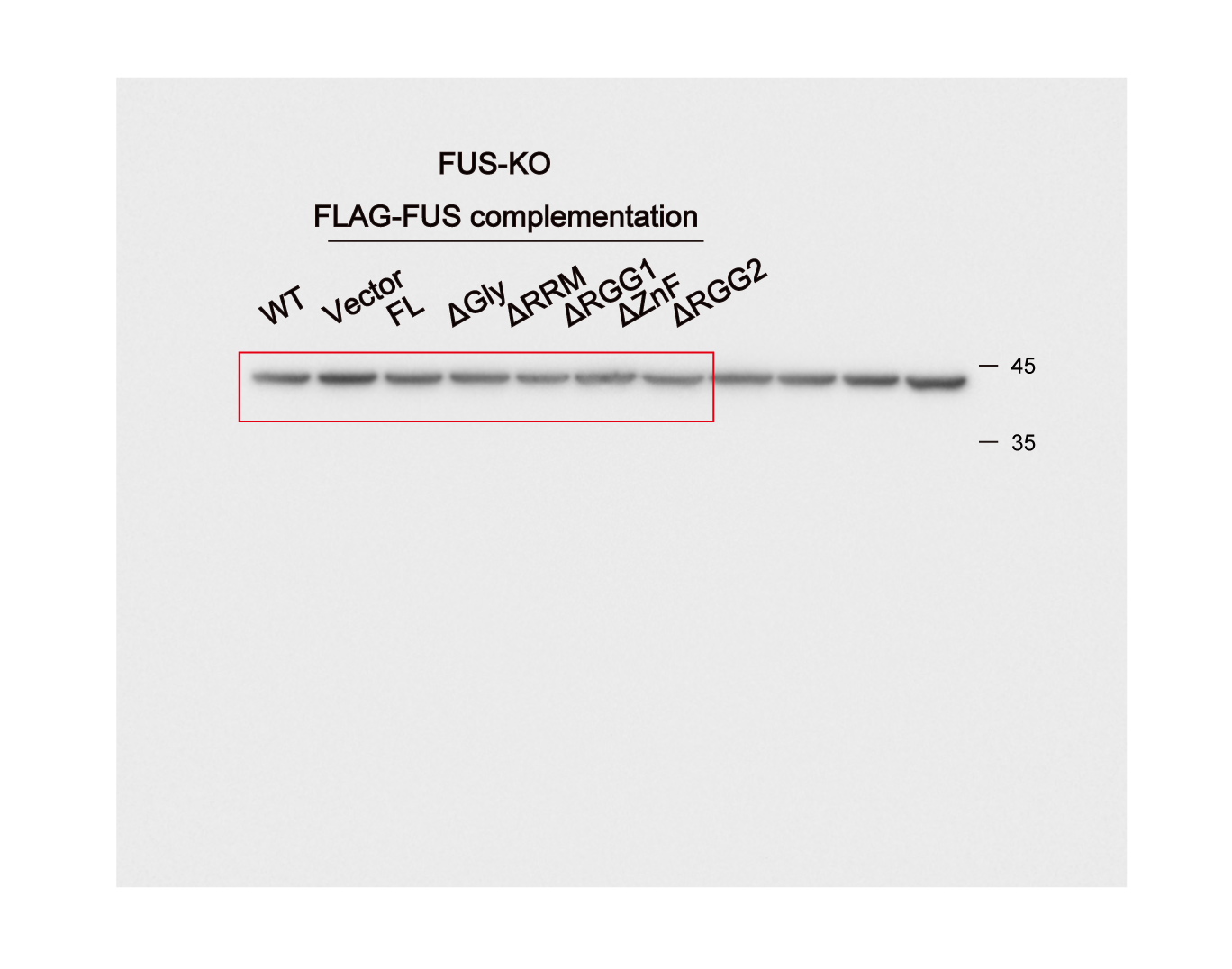

Supplement: Supplementary file 11 — Source data Fig. 6 [file 44318_2025_506_MOESM11_ESM.zip › Figure 6/Fig6B/Western_beta-actin.tif]

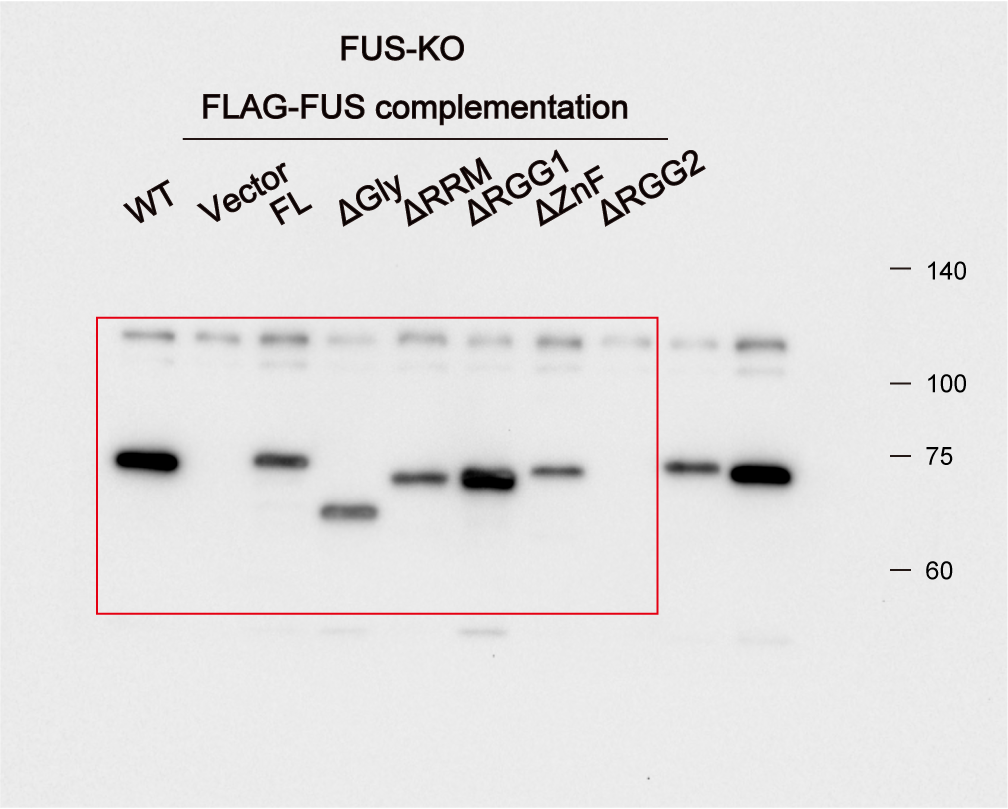

Supplement: Supplementary file 11 — Source data Fig. 6 [file 44318_2025_506_MOESM11_ESM.zip › Figure 6/Fig6B/Western_FUS.tif]

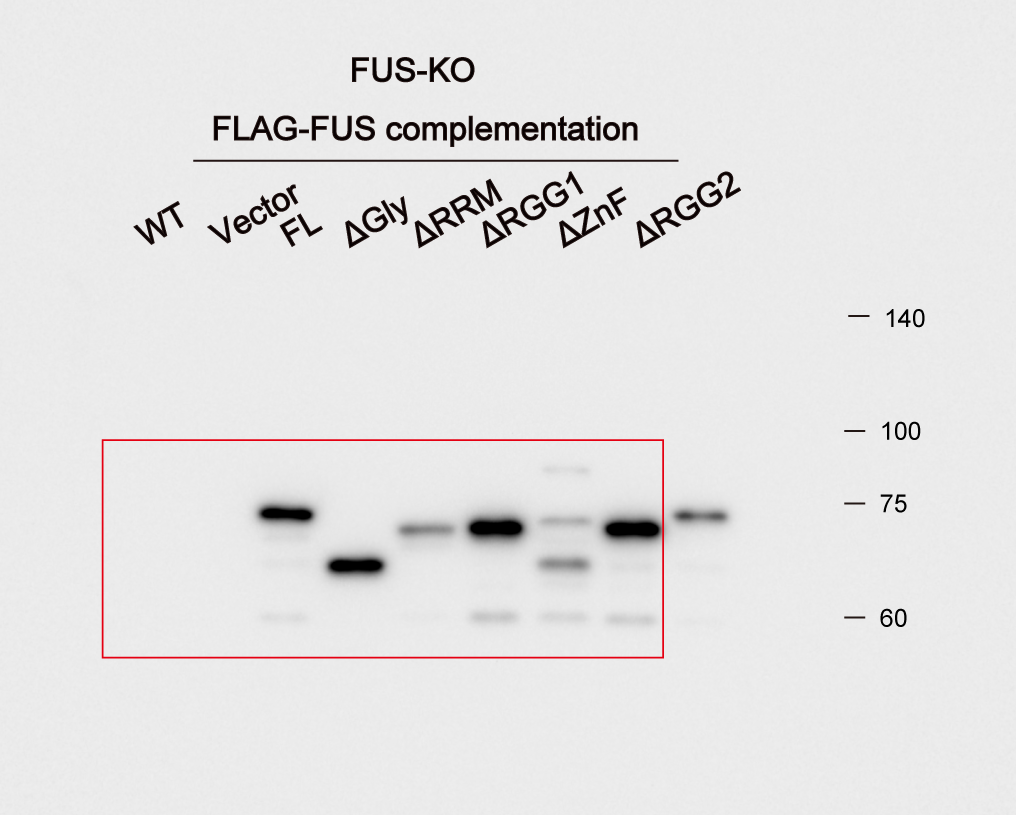

Supplement: Supplementary file 11 — Source data Fig. 6 [file 44318_2025_506_MOESM11_ESM.zip › Figure 6/Fig6B/Western_hnRNPA1.tif]

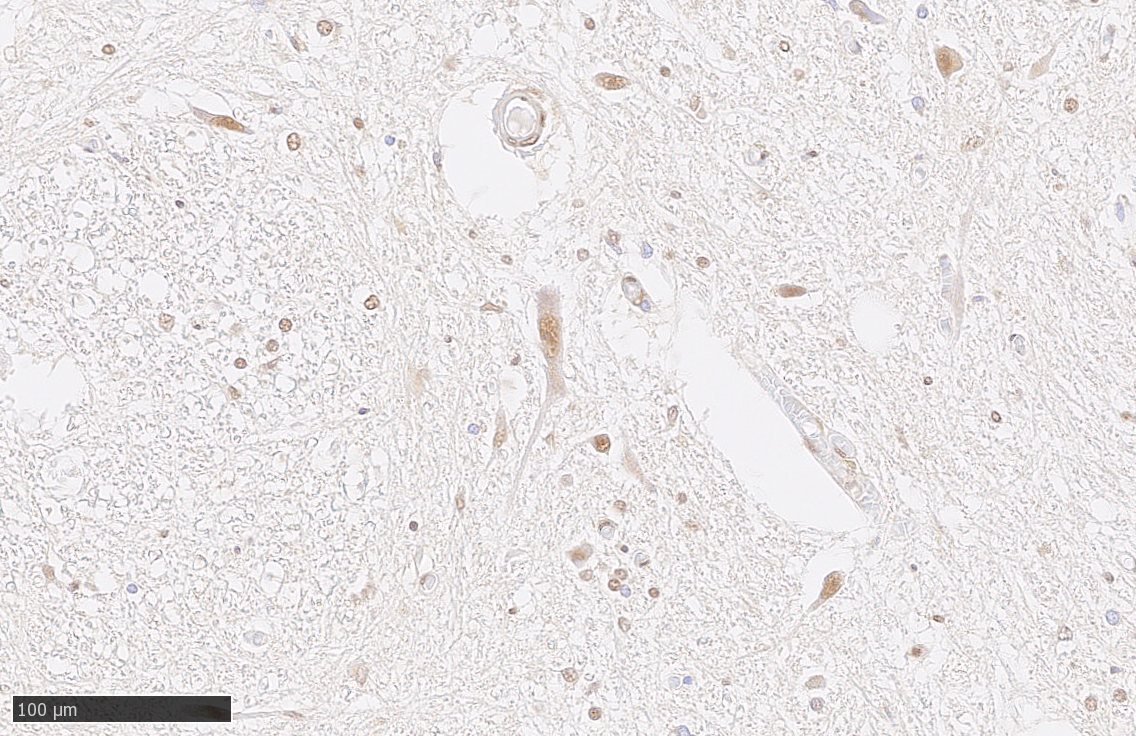

Supplement: Supplementary file 12 — Source data Fig. 7 [file 44318_2025_506_MOESM12_ESM.zip › Figure 7/Fig7H/Original_Fig7H.tif]

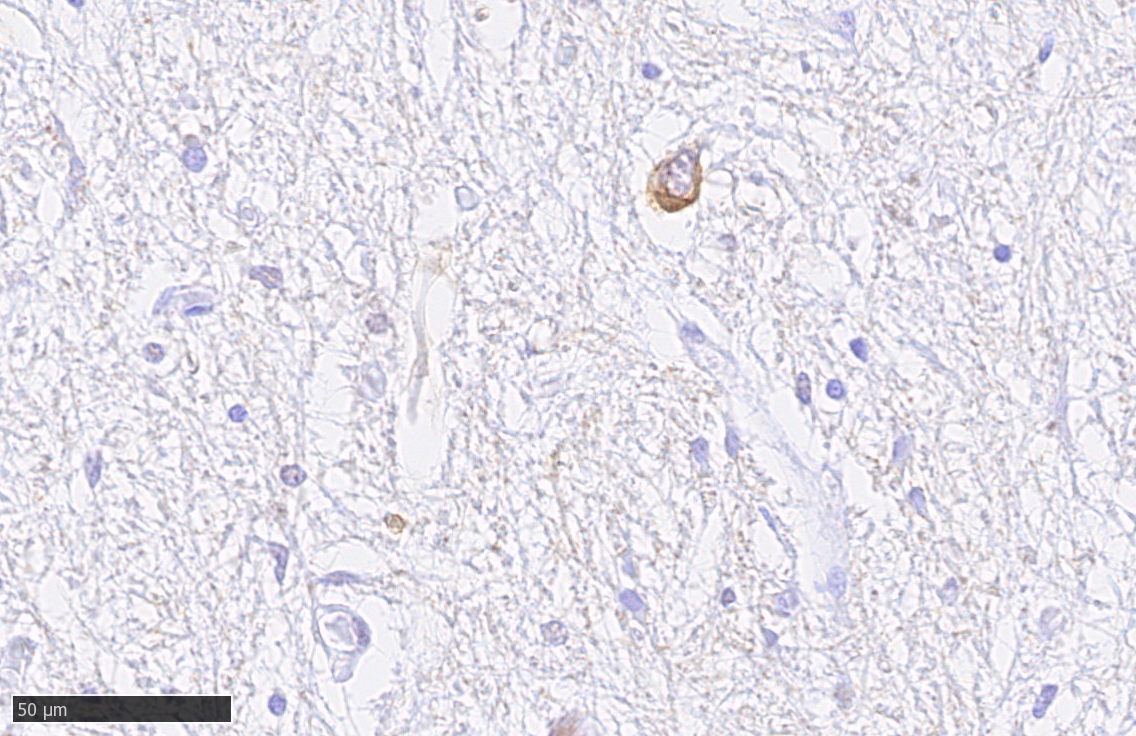

Supplement: Supplementary file 12 — Source data Fig. 7 [file 44318_2025_506_MOESM12_ESM.zip › Figure 7/Fig7F/Original_Fig7F.tif]

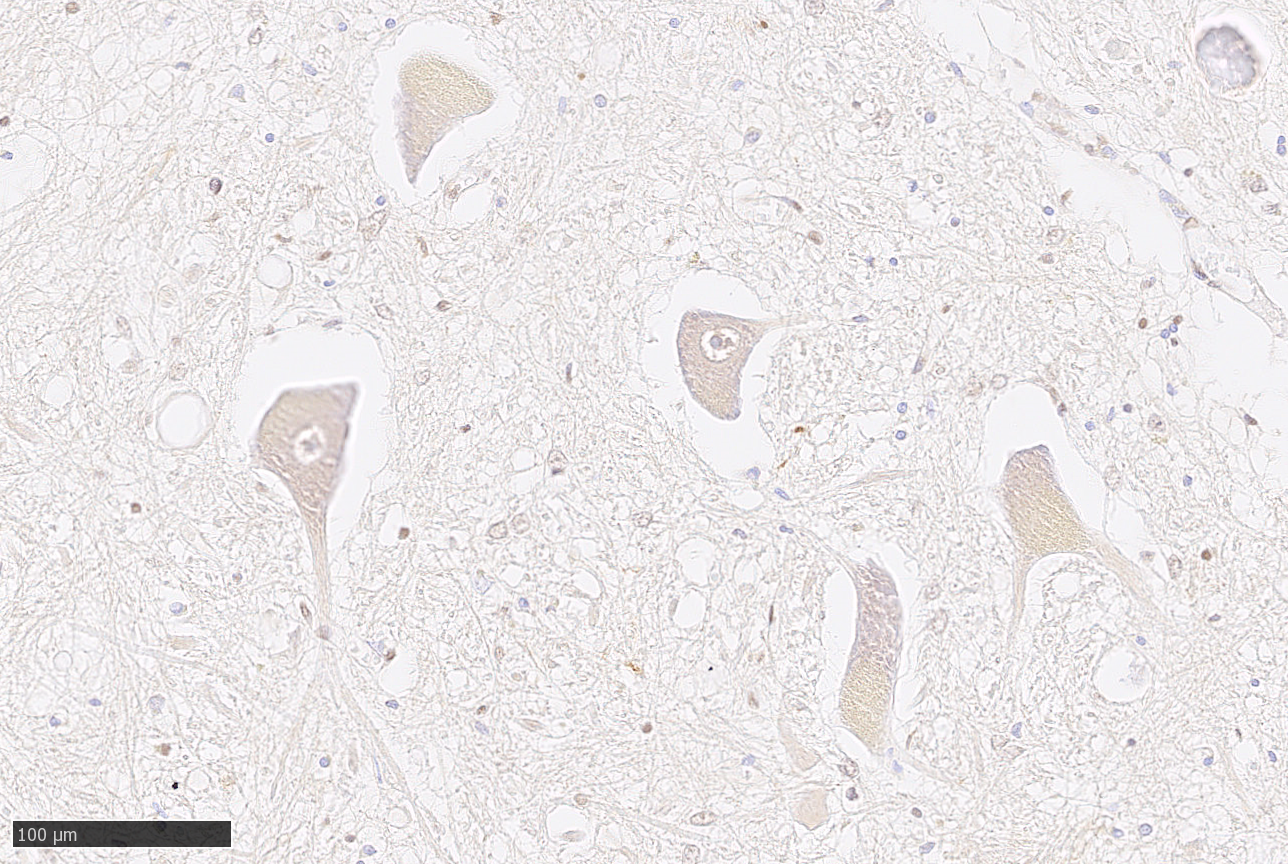

Supplement: Supplementary file 12 — Source data Fig. 7 [file 44318_2025_506_MOESM12_ESM.zip › Figure 7/Fig7G/Original_Fig7G.tif]

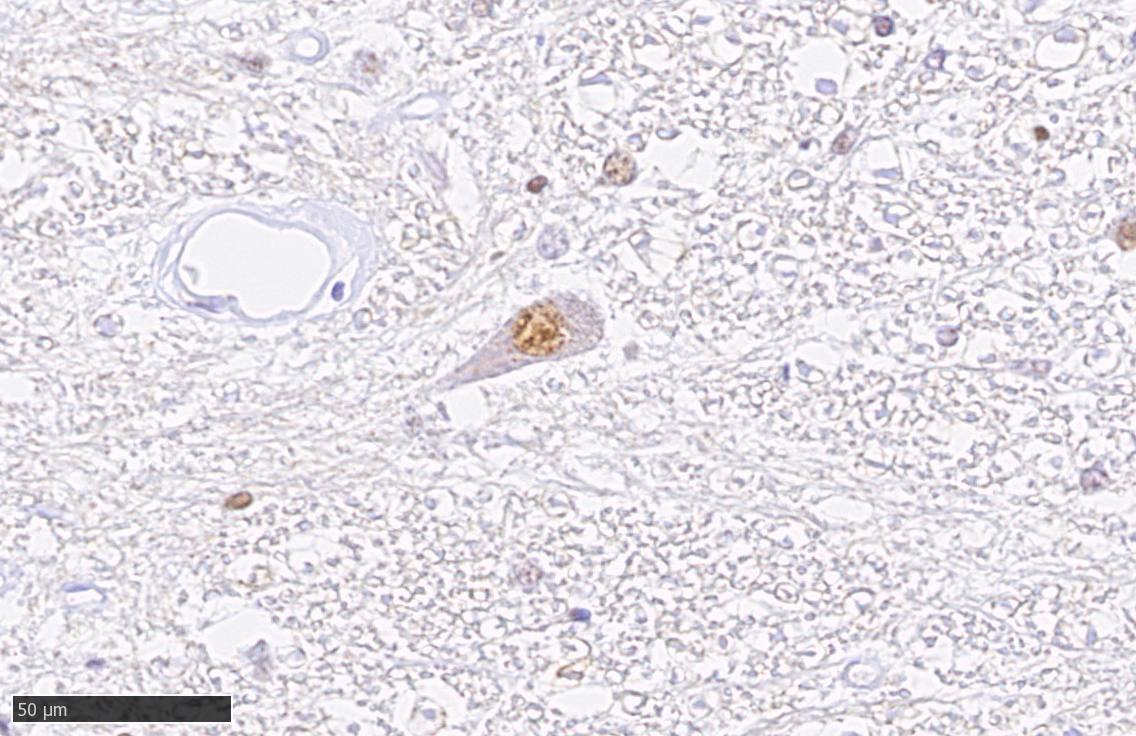

Supplement: Supplementary file 12 — Source data Fig. 7 [file 44318_2025_506_MOESM12_ESM.zip › Figure 7/Fig7E/Original_Fig7E.tif]

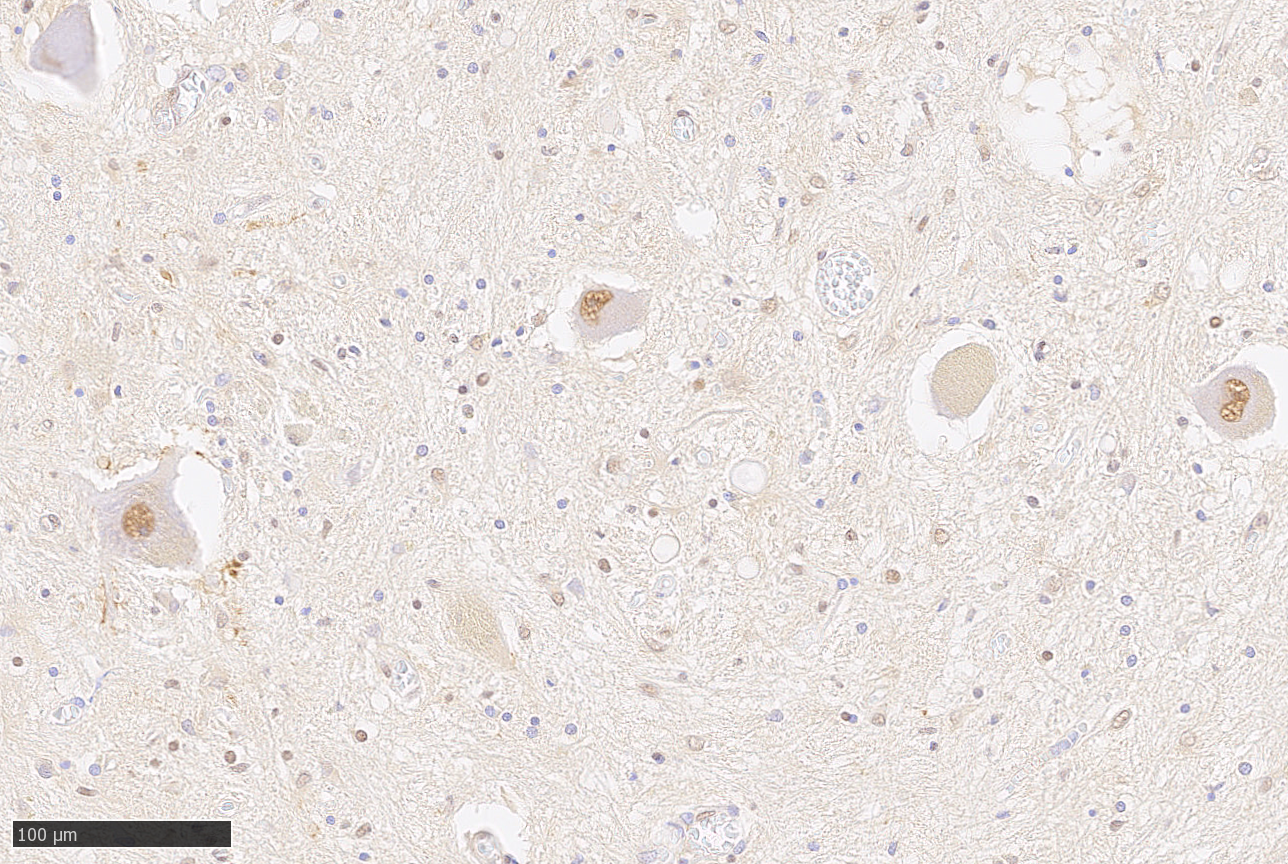

Supplement: Supplementary file 12 — Source data Fig. 7 [file 44318_2025_506_MOESM12_ESM.zip › Figure 7/Fig7J/ALS(b).tif]

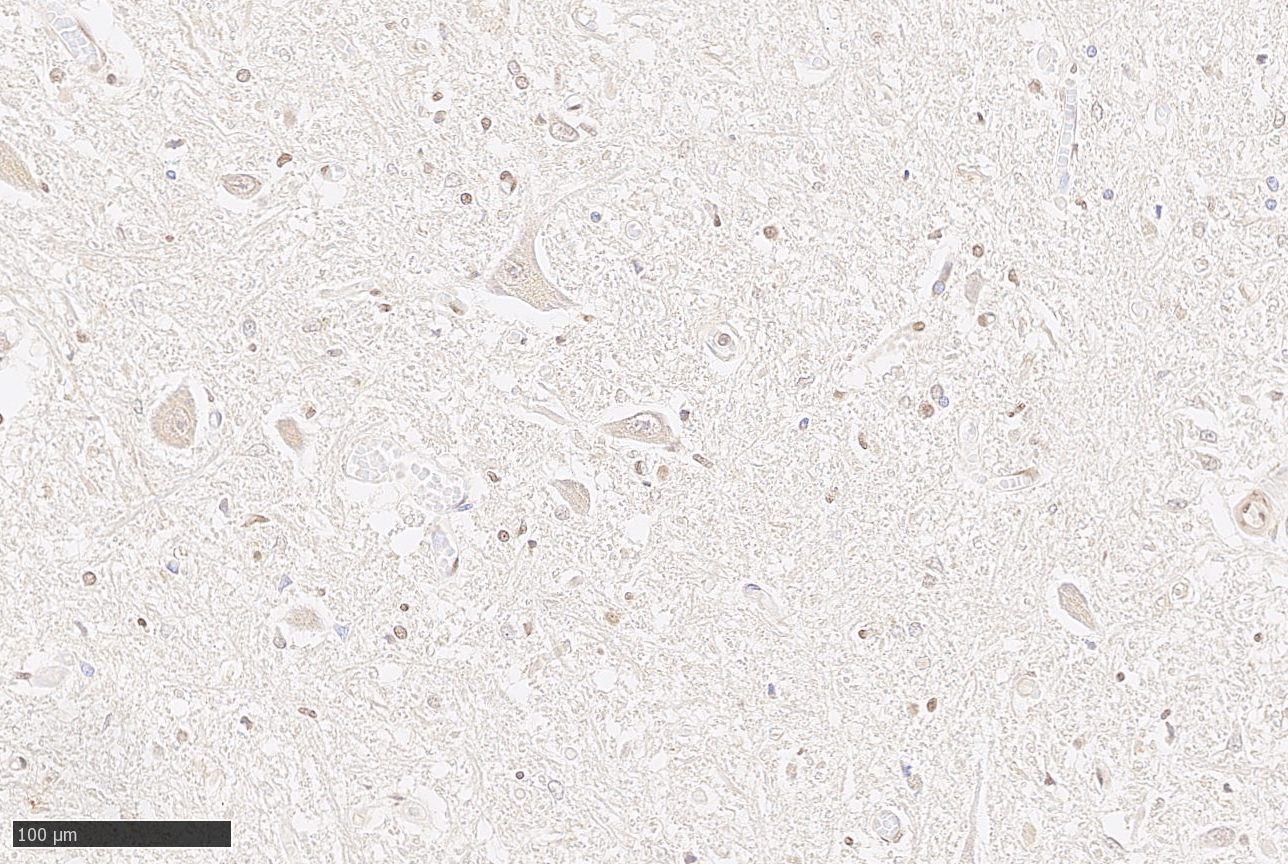

Supplement: Supplementary file 12 — Source data Fig. 7 [file 44318_2025_506_MOESM12_ESM.zip › Figure 7/Fig7J/Non-ALS3.tif]

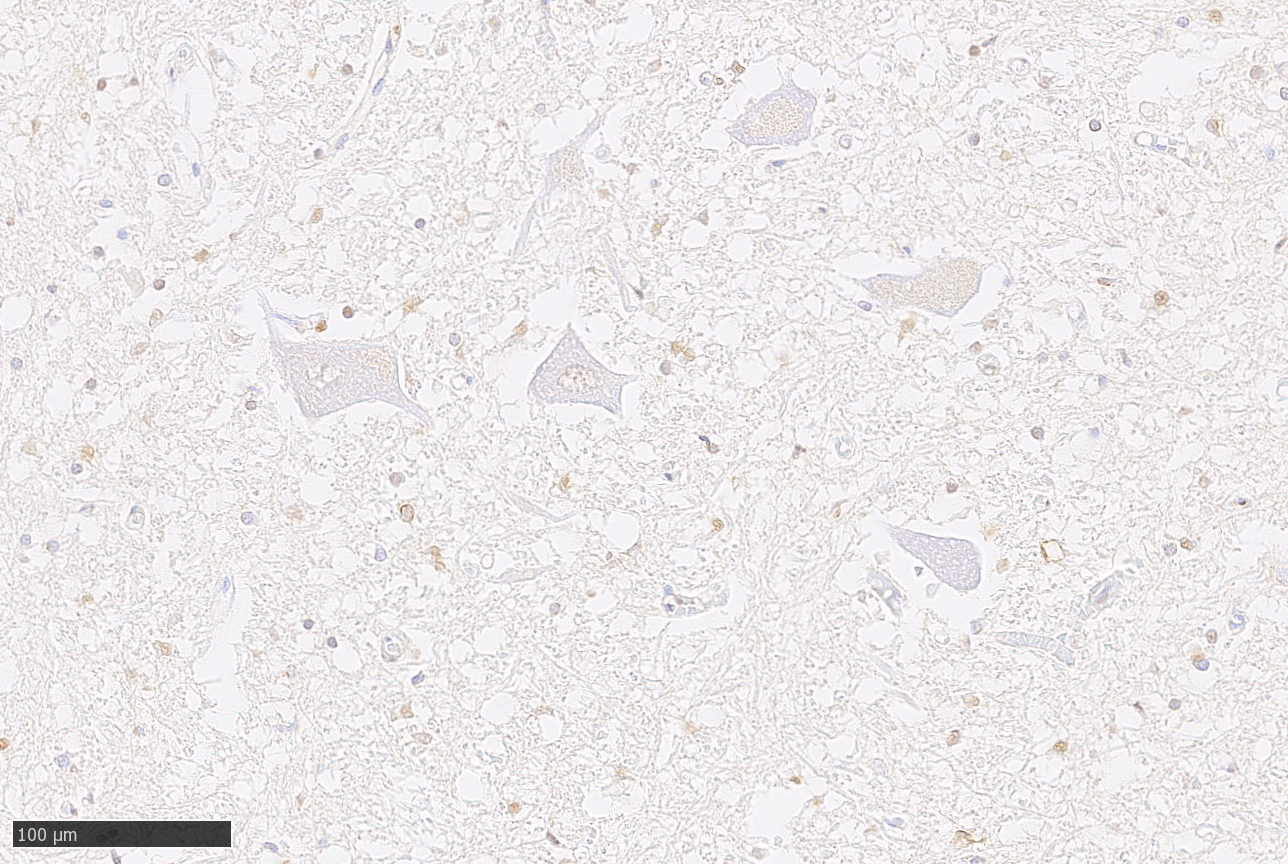

Supplement: Supplementary file 12 — Source data Fig. 7 [file 44318_2025_506_MOESM12_ESM.zip › Figure 7/Fig7J/Non-ALS2.tif]

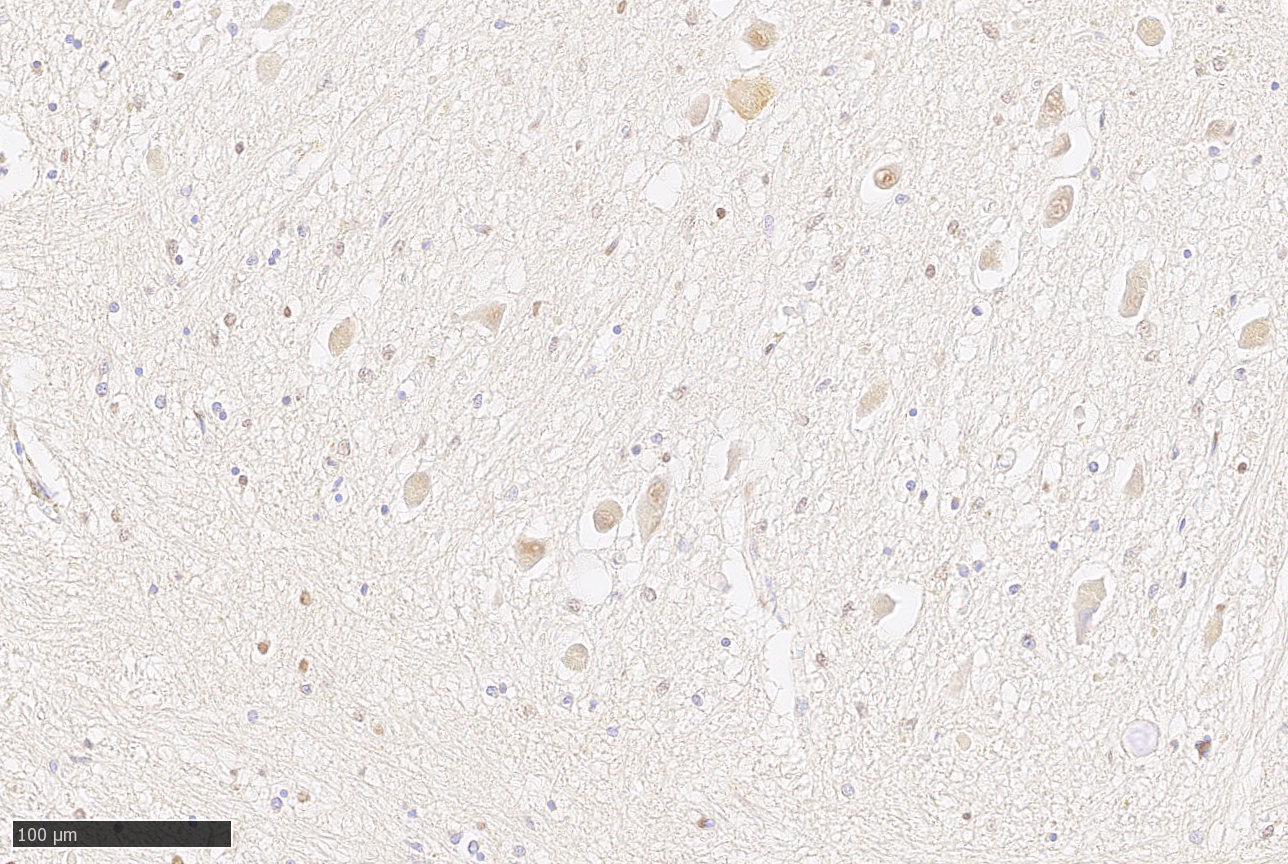

Supplement: Supplementary file 12 — Source data Fig. 7 [file 44318_2025_506_MOESM12_ESM.zip › Figure 7/Fig7J/ALS(c).tif]

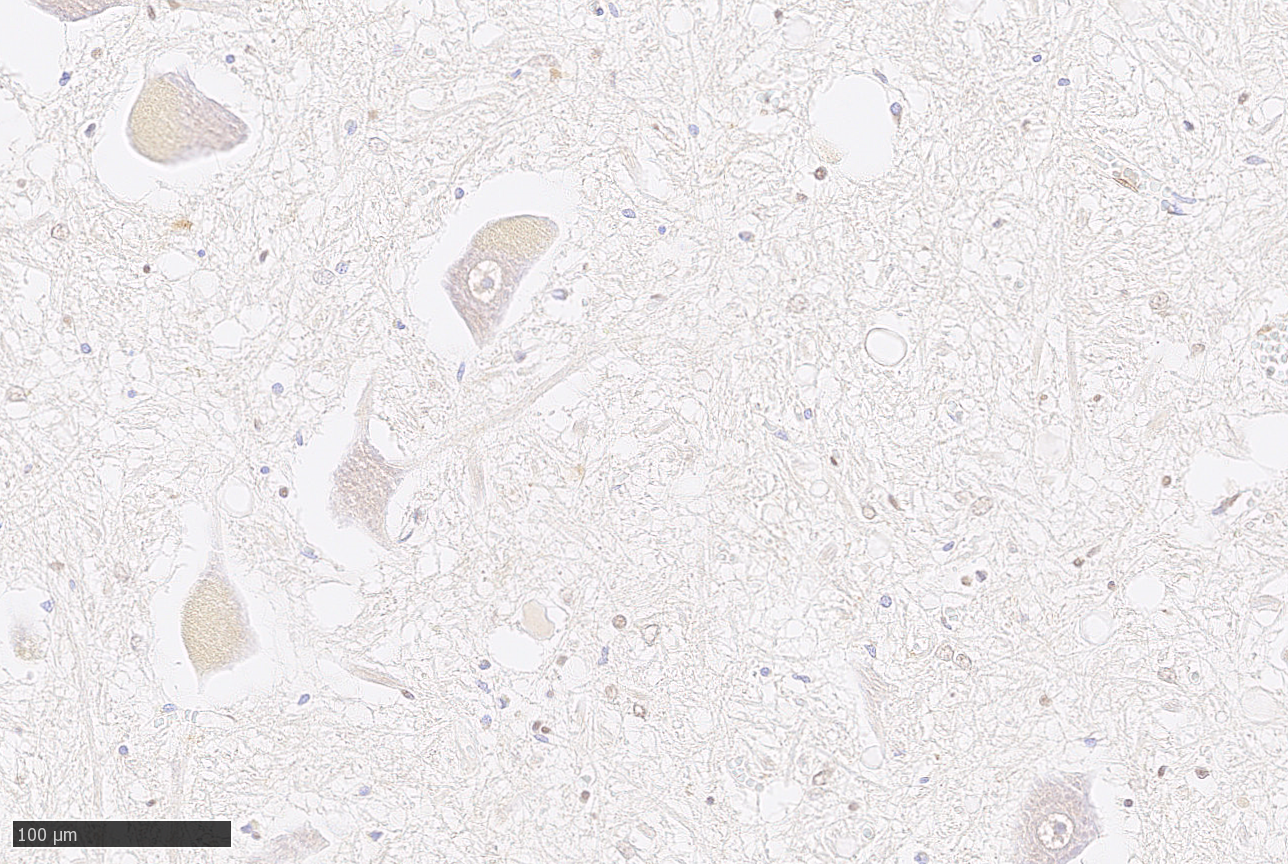

Supplement: Supplementary file 12 — Source data Fig. 7 [file 44318_2025_506_MOESM12_ESM.zip › Figure 7/Fig7J/Non-ALS1.tif]

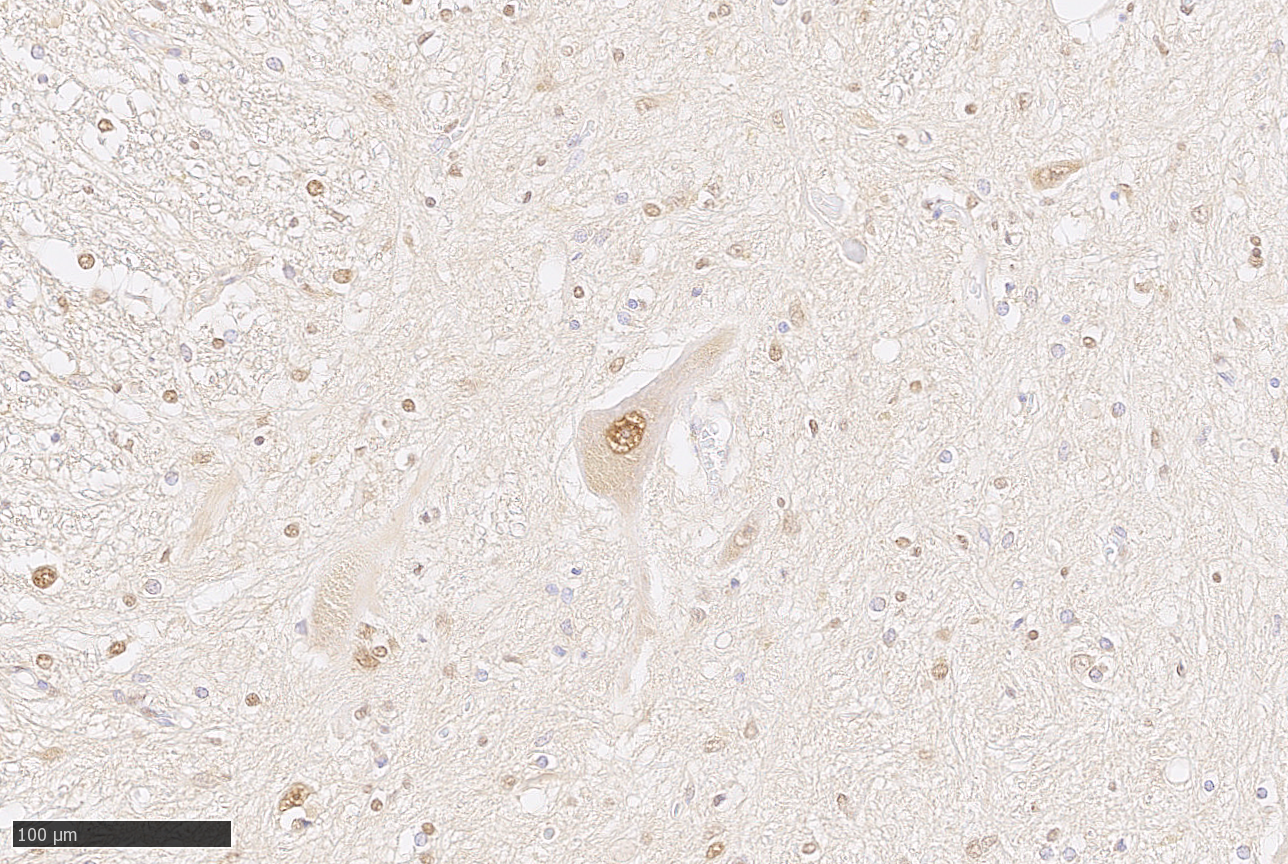

Supplement: Supplementary file 12 — Source data Fig. 7 [file 44318_2025_506_MOESM12_ESM.zip › Figure 7/Fig7J/ALS(a).tif]
