# Supplementary material for: ALS-associated RNA-binding proteins promote UNC13A transcription through REST downregulation
Source: EMBO J. 2025 Jul 24;44(17):4745–71. doi: 10.1038/s44318-025-00506-0 (PMC12402202; doi:10.1038/s44318-025-00506-0)
Supplement: Supplementary file 13 — Expanded View Figures [file 44318_2025_506_MOESM13_ESM.pdf]

Expanded View Figures

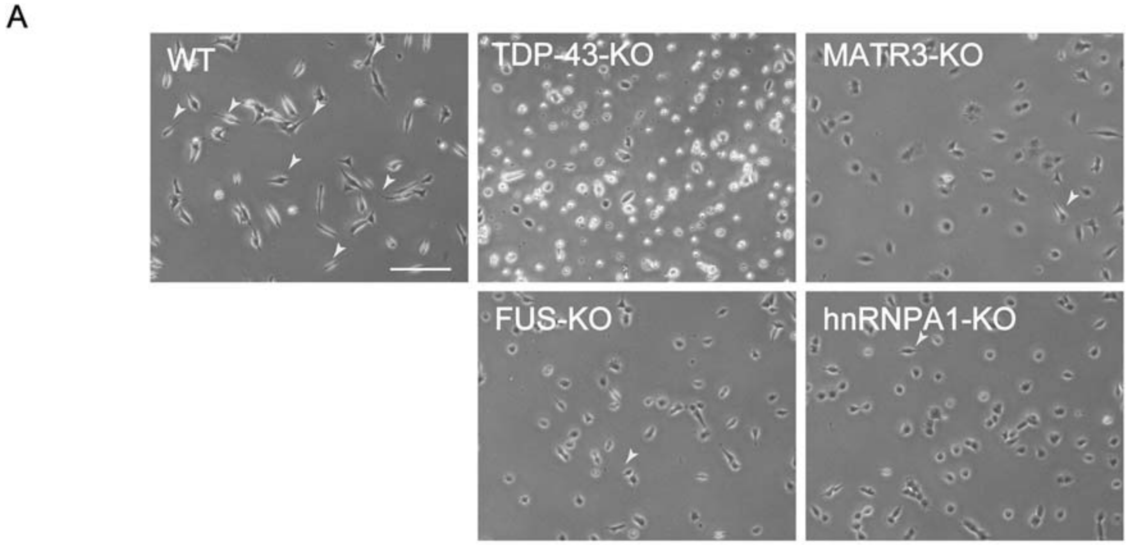

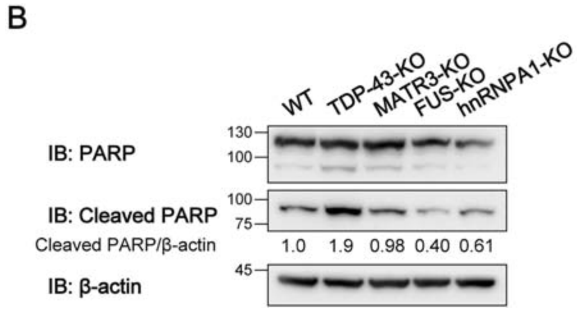

Figure EV1. Characterization of RBP-KO cell lines, related to Fig. 1.

(**A**) Bright-field images of WT cells and RBP-KO cell lines. Cells were seeded in iMatrix-coated wells, and images were captured the next day. Yellow arrowheads indicate cells with cytoplasmic processes. Scale bar = 200 μm. (**B**) Immunoblot analysis of PARP and cleaved PARP in WT cells and in RBP-KO cell lines. β-actin served as a loading control. The band intensity for cleaved PARP (normalized by that of β-actin) was quantified by densitometry.

© The Author(s)

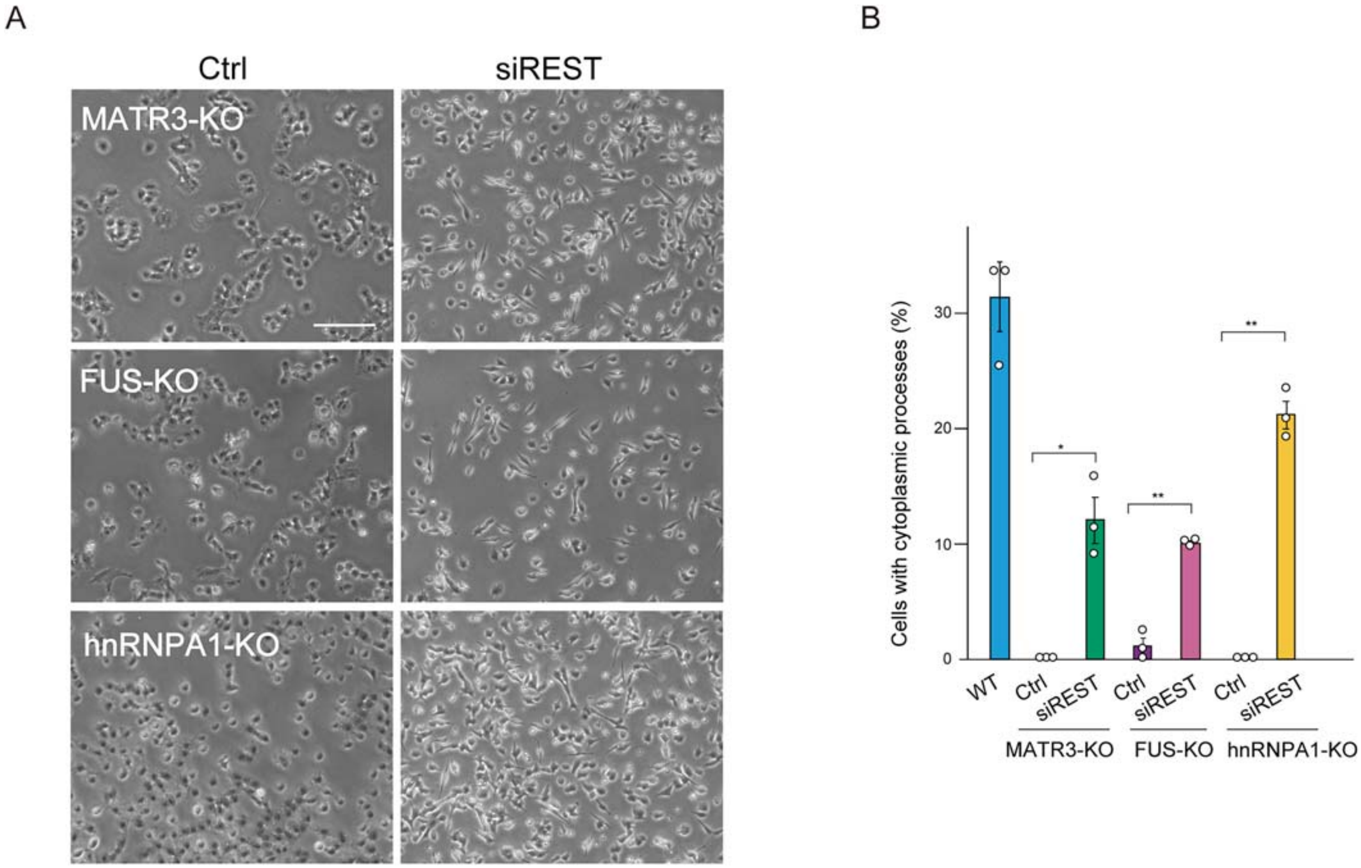

Figure EV2. Knockdown of REST rescues morphological defects in RBP-KO cell lines, related to Fig. 3.

(**A**) Bright-field images of MATR3-, FUS-, and hnRNPA1-KO cell lines in iMatrix-coated 6-well plate, 5 days after transfection with either a GC duplex (negative control) or a *REST* siRNA. Scale bar = 200 μm. (**B**) Quantification of cytoplasmic process formation in MATR3-KO, FUS-KO, and hnRNPA1-KO cell lines shown in (**A**), with WT cells included as a reference. Cells were classified as having cytoplasmic processes if their extensions exceeded the length of the major axis of the cell body. Data are mean ± SEM from three independent experiments. $^{**}P < 0.01$, $^{***}P < 0.001$, $^{****}P < 0.0001$ (Student's *t* test); exact *P* values: MATR3-KO, $P = 0.0036$; FUS-KO, $P = 0.00027$; hnRNPA1-KO, $P = 6.6\text{e-}05$.

 © The Author(s)

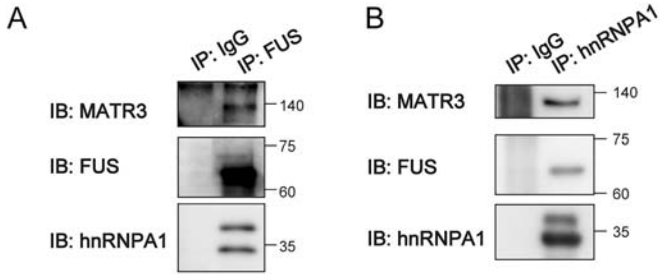

Figure EV3. FUS, MATR3, and hnRNPA1 bind to each other, related to Fig. 5.

(**A**) Immunoprecipitation of FUS from WT cell lysate. The immunoprecipitate (IP) obtained with antibodies to FUS or with control immunoglobulin G (IgG) were subjected to immunoblot analysis with antibodies to MATR3, FUS or hnRNPA1. (**B**) Immunoprecipitation of hnRNPA1 from WT cell lysate.

© The Author(s)

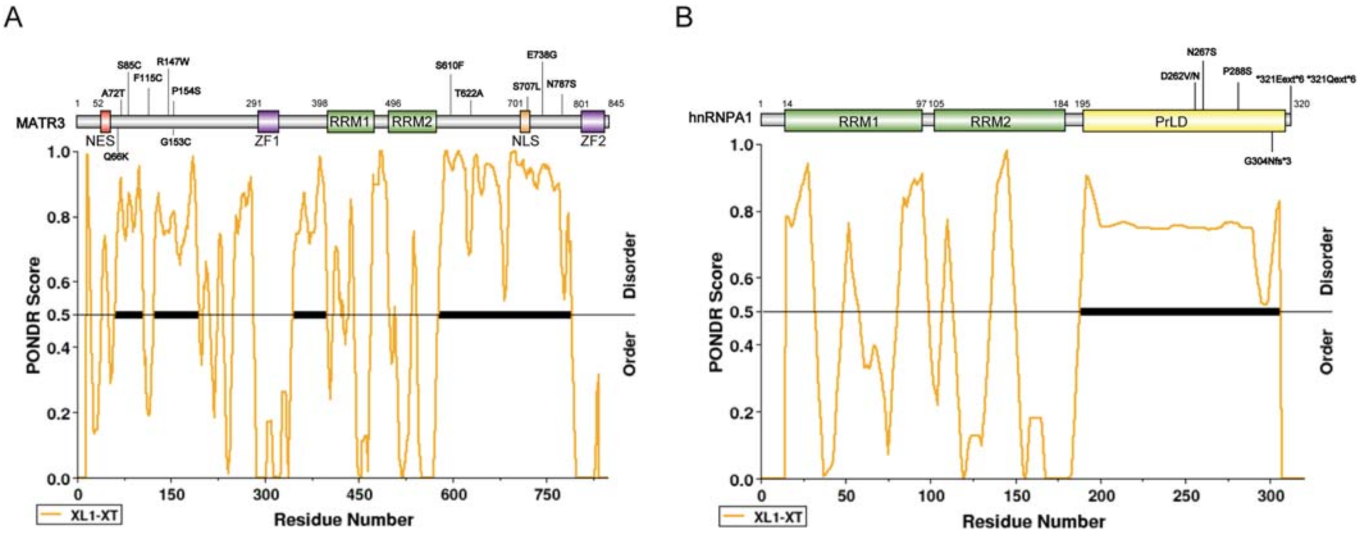

Figure EV4. Domain structures of MATR3 and hnRNPA1 with ALS-associated mutations, related to Fig. 6.

(**A**) Schematic representation of the domain structure of MATR3 (top). NES, nuclear export signal; ZnF, zinc finger; RRM, RNA recognition motif; NLS, nuclear localization signal. The locations of ALS-associated mutations in MATR3 referenced from Malik and Barmada, 2021 are also shown. Disorder prediction for MATR3 residues by PONDR (http://www.pondr.com) is shown at the bottom. (**B**) Schematic representation of the domain structure of hnRNPA1 (top). RRM, RNA recognition motif; PrLD, prion-like domain. The locations of ALS-associated mutations in hnRNPA1 referenced from Beijer et al, 2021 are also shown. G304Nfs*3 is a frameshift mutation, while *321Eext*6 and *321Qext*6 are extension mutations. Disorder prediction for hnRNPA1 residues by PONDR is shown at the bottom.

© The Author(s)

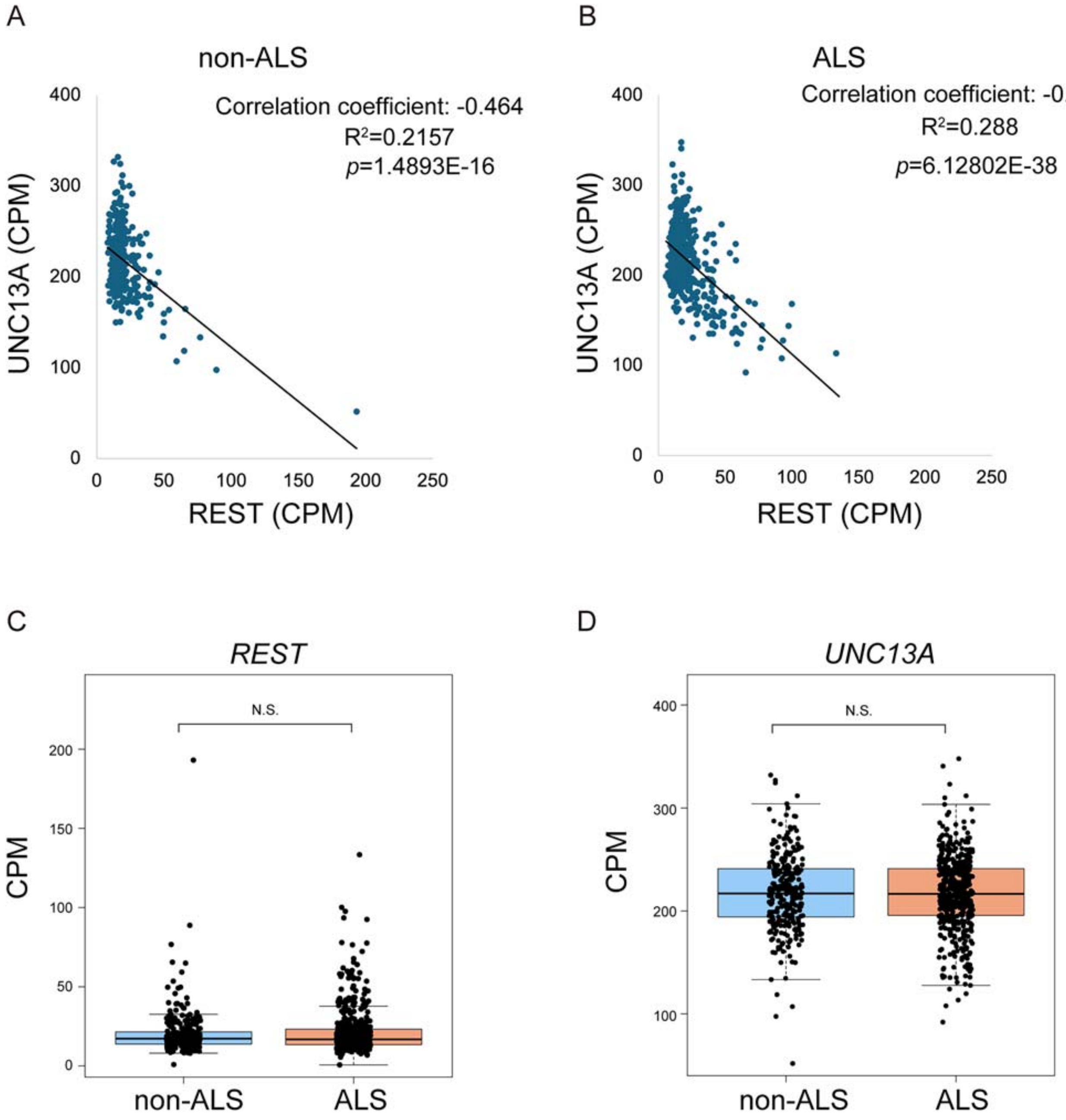

Figure EV5. *REST* and *UNC13A* expression in iPSC-derived motor neurons from the Answer ALS platform, related to Fig. 7.

(**A**, **B**) Correlation analysis of *REST* and *UNC13A* expression using transcriptomics data from iPSC-derived motor neurons available on the Answer ALS platform. Each blue dot represents one sample. The line indicates the linear regression fit. The R^2 value represents the proportion of variance in *UNC13A* expression explained by REST expression, and the *p* value was calculated using Pearson's correlation test. (**A**) Non-ALS group ($n = 283$), (**B**) ALS group ($n = 490$). CPM, counts per million. (**C**, **D**) Comparison of *REST* (**C**) and *UNC13A* (**D**) expression between control (non-ALS, $n = 283$) and ALS (ALS, $n = 490$) using transcriptomics data from iPSC-derived motor neurons available on the Answer ALS platform. Box plots represent the median (center line), interquartile range (IQR; box), and whiskers indicating the most extreme data point within 1.5×IQR from the quartiles. CPM, counts per million. N.S., not significant (Mann–Whitney *U* test).

© The Author(s)
